# Review of Deep Learning Approaches for Interleaved Photoacoustic and Ultrasound (PAUS) Imaging

**DOI:** 10.1109/TUFFC.2023.3329119

**Published:** 2023-12-14

**Authors:** MinWoo Kim, Ivan Pelivanov, Matthew O’Donnell

**Affiliations:** Department of Biomedical Convergence Engineering and the Center for Artificial Intelligence Research, Pusan National University, Busan 46241, South Korea; uWAMIT Center, Department of Bioengineering, University of Washington, Seattle, WA 98105 USA; uWAMIT Center, Department of Bioengineering, University of Washington, Seattle, WA 98105 USA

**Keywords:** Deep learning (DL), image reconstruction, neural network, photoacoustic (PA) imaging, PA and ultrasound (PAUS)

## Abstract

Photoacoustic (PA) imaging provides optical contrast at relatively large depths within the human body, compared to other optical methods, at ultrasound (US) spatial resolution. By integrating real-time PA and US (PAUS) modalities, PAUS imaging has the potential to become a routine clinical modality bringing the molecular sensitivity of optics to medical US imaging. For applications where the full capabilities of clinical US scanners must be maintained in PAUS, conventional limited view and bandwidth transducers must be used. This approach, however, cannot provide high-quality maps of PA sources, especially vascular structures. Deep learning (DL) using data-driven modeling with minimal human design has been very effective in medical imaging, medical data analysis, and disease diagnosis, and has the potential to overcome many of the technical limitations of current PAUS imaging systems. The primary purpose of this article is to summarize the background and current status of DL applications in PAUS imaging. It also looks beyond current approaches to identify remaining challenges and opportunities for robust translation of PAUS technologies to the clinic.

## Graphical Abstract

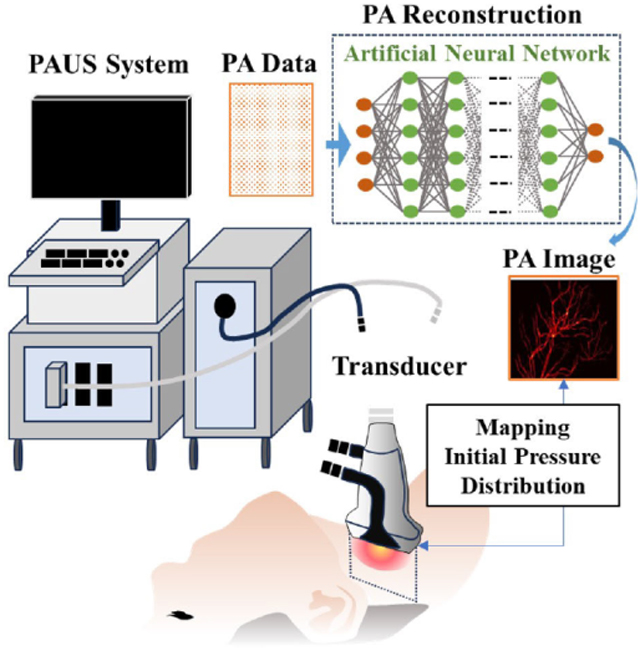

## I. Introduction

Photoacoustic (PA) imaging combines optical contrast with ultrasound (US) image formation. It exploits the PA effect in which time-modulated light is absorbed in chromophores within biological tissue, inducing differential thermoelastic expansion forming acoustic waves (or PA signals) [1], [2]. The common imaging framework is to emit a sequence of nanosecond laser pulses into the tissue region of interest (ROI) and detect induced PA signals using an array of acoustic sensors [3], [4], [5]. The primary advantage of this approach compared to other pure optical methods is that light diffusion within tissue does not affect PA image resolution at any depth because it is entirely defined by US (acoustic resolution). This enables optical detection of vascular diseases and cancers or monitoring physiological changes in relatively deep tissue areas [6], [7], [8] through PA imaging of light absorption. Combined with medical US, PA imaging brings a molecular dimension using targeted molecular contrast agents such as dyes and various nanoparticles [9], [10].

Many PA systems have been developed that optimize the laser source, irradiation geometry, and sound detectors for a specific application [11], [12], [13], [14], [15], [16], [17], [18], [19], [20], [21], [22]. Among them, PA-computed tomography (PACT) reconstructs a macroscopic image with relatively deep penetration (a few centimeters) by strategically combining PA signals emitted from the entire ROI and detected by multiple sensors [23], [24]. Specifically, a broadened laser beam in tissue can access a large ROI for a short time, and a sensor array can acquire PA signals over time at different positions for each laser firing. The universal back-projection (UBP) algorithm derived from the spherical Radon transform reconstructs a quantitative map of optical absorption from PA measurements provided that the detection array is full view and full bandwidth [25], the laser fluence distribution in the medium is known, and the medium is acoustically and thermally homogeneous. PACT generally uses a hemisphere or cylindrical sensor array to surround a target, so the main applications have been small animal imaging or monitoring human breast disease [19].

**Fig. 1.**
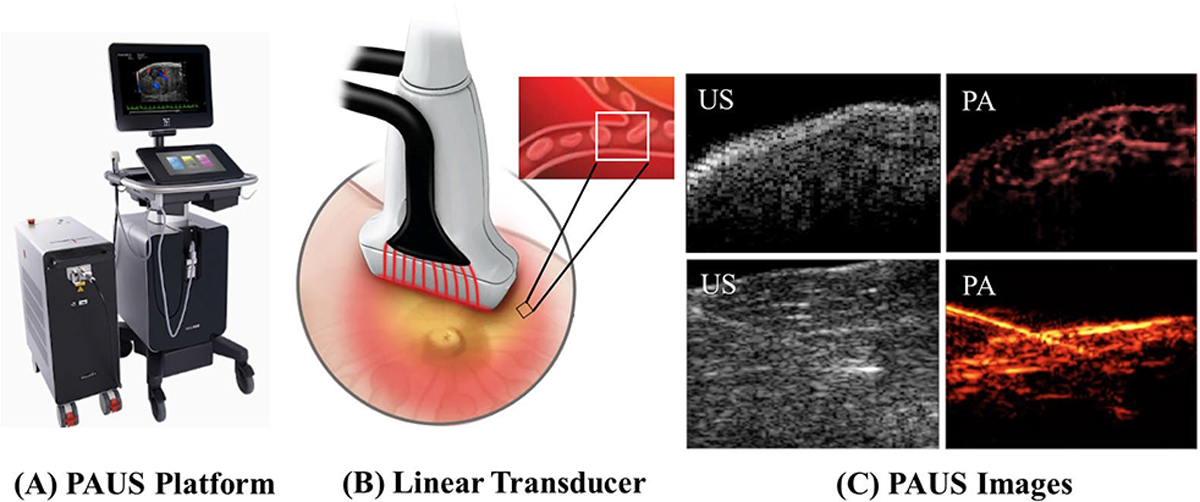
(a) One example of a commercial PAUS platform Vevo LAZR-X, Visualsonics). (b) Optical fibers are located at/near the surface of the clinical US transducer to deliver laser energy into tissue (linear transducer). The PA signal is acquired by the transducer’s piezoelectric sensors. (c) US and PA images. The main target is microvessels, or endogenous/exogenous molecules in image-guided interventions. Top images show small vessels in a human finger and bottom images show a needle insertion and gold nanorod injection into chicken breast tissue. (b) and (c) are reproduced with permission from [26] and [27].

Although nearly ideal for small animal studies, the PACT model is limited for general clinical applications by the array geometry. Due to limited acoustic access, as well as cost and physical complexity, large-scale hemispherical and cylindrical arrays are impractical for most clinical applications. An alternative approach is to integrate an optical delivery system within a standard US scanner for interleaved, real-time PAUS imaging [16], [26], [28], [29], [30], [31]. A handheld US transducer contains a piezoelectric sensor array. For conventional 1-D arrays providing real-time 2-D images, optical fibers or light-emitting diode (LED) sources [32], [33] are located near/on the transducer surface to deliver laser light into the tissue volume (see Fig. 1). Since the transducer is planar or almost planar (typically convex), unlike the circular geometry, users can flexibly position it on any surface of the body. Laser and fiber delivery systems and scanning protocols have been developed to generate simultaneous US and PA images at real-time imaging rates (>20 Hz) for clinical use [26], [29].

The primary advantage of the PAUS approach is that it leverages not only PA imaging but also the current state of the art in real-time US. Thus, it is appropriate for a number of clinical applications and is particularly well suited for image-guided interventional procedures, such as drug delivery and surgeries, where PA imaging provides a molecular dimension missing from current US guidance systems [34], [35]. Some companies have already released commercial PAUS scanners for animal studies, which display real-time PA and conventional US images. Many academic research groups have also implemented PAUS systems by simply modifying a commercial US platform. Schellenberg and Hunt [36] specifically reviewed such systems and associated clinical trials.

However, PAUS imaging has not yet been routinely adopted for human applications in the clinic. The critical hurdles are still low image quality and inaccurate quantitative measures. Specifically, PA image reconstruction from raw sensor data represents a severely ill-posed problem due to the limited view and narrow bandwidth of clinical US transducers [37], [38]. As a result, the PA image is far from a one-to-one map of optical absorbers, and a range of diverse artifacts complicates image interpretation. These images are further degraded by common acoustic issues such as reverberations or clutter, reflection artifacts, and speed of sound (SOS) aberrations [39], [40], [41].

Designing a transducer array simultaneously optimizing PA and US imaging poses significant challenges given the practical constraints of handheld operation. In medical US, to effectively convert electrical power to acoustic waves and form transmit acoustic beams, US transducers must be relatively narrowband. As a consequence, US B-mode images exhibit speckle due to local heterogeneities in US scattering. A large transducer aperture and a broad view are not usually required, and for most applications, the transducer should be quite compact to enable access to different organs within the human body.

In contrast, a PA image is formed by the distribution of heat release in the medium induced by pulsed laser irradiation. Reconstructing the spatial distribution of heat release is mathematically very different from reconstructing a local scattering function. It requires a detection system with ultrabroadband detection and a geometry that captures all potential propagation paths from sources (i.e., full view). Consequently, the optimal detection configurations are very different for US and PA modalities.

Recent studies have explored a hemispherical handheld array [42] to enhance PA image quality using tomographic reconstruction, but this probe is also suboptimal for conventional US imaging due to its limited effective field of view [36], [42]. Standard US probes (transducers) are typically designed as linear or convex arrays, taking various factors into account, including not only the scan view but also cost, image quality, scanning convenience, and clinical applications. We believe that for widespread adoption of PAUS systems, the probe and system must be optimized for high-quality, real-time US imaging. This means that PA image quality will be sacrificed. If the transducer characteristics and geometry cannot be optimized in the PAUS configuration, advanced reconstruction methods that can compensate for the transducer’s limited view and bandwidth are in high demand. Consequently, PA advanced reconstruction through data processing could potentially improve overall PAUS image quality to a level enabling largescale clinical applications such as procedure guidance. For instance, it can assist in guiding drug release to an optimal target position, ablation procedures, and biopsy needles [43], [44].

Deep learning (DL) techniques [45], [46], [47] have significantly impacted biomedical imaging in areas such as microscopy [12], histology [48], MRI [49], [50], and CT [51], [52]. They also have the potential to address the primary limitations of PAUS imaging because of their strong generalizability and efficiency. To handle ill-conditioned problems, standard mathematical or handcrafted models require additional human knowledge, specific hypotheses, and/or physical phenomena that are often difficult to generalize for all data acquisition environments. In contrast, DL is a data-driven approach without priors that can be trained with many plausible data samples to capture the essential features of real cases.

Currently, many neural network types have demonstrated their superior ability to adapt to new data [53]. In addition, computational time is much lower compared to standard model-based techniques depending on iterative schemes. Thus, the core procedure is: 1) develop a concise DL network to automatically extract features that reduce data redundancy and narrow possible solutions against ill-posed conditions and 2) create optimal training samples to guide the network to adapt to a wide distribution of real samples.

Overall, DL studies for PA imaging were well summarized in [54], [55], [56], [57], and [58]. They encompassed various DL applications, including image reconstruction, image understanding (classification and segmentation), and quantitative imaging. In this article, we focus on imaging (specifically the reconstruction of initial pressure images) in the geometry provided by clinical US that enables PAUS imaging using handheld probes and conceptually review current work exploiting DL frameworks to overcome fundamental PAUS limitations. Specifically, in Section II, we outline the PA signal acquisition geometry and standard image reconstruction procedure. Section III describes DL work, including in-silico, in vitro, or in vivo data generation and neural network construction to process these data. Section IV summarizes the findings and discusses remaining challenges and opportunities.

## II. Photoacoustic Imaging

**Fig. 2.**
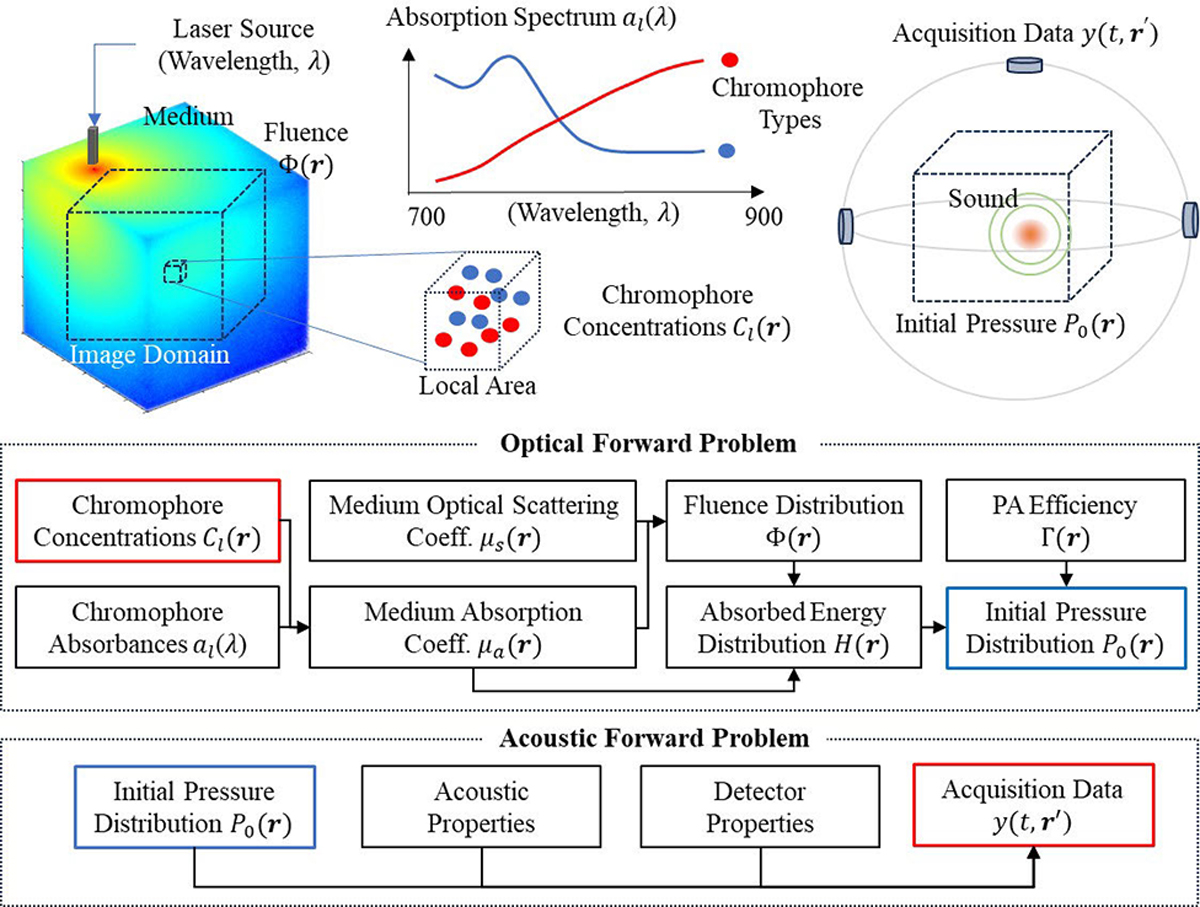
Two processes drive PA signal acquisition. First, the optical forward process describes the generation of initial pressure derived from chromophore concentrations and the light distribution (fluence) within the 3-D medium. Second, the acoustic forward process describes the acquisition of acoustic waves originating from the initial pressure. The ultimate goal of PA imaging is to accurately quantify chromophore concentrations from acquired data. In general, two steps are required to solve this inverse problem. First, the initial pressure distribution is reconstructed by addressing the acoustic inverse problem. Then, chromophore concentrations are estimated by solving the optical inverse problem using the pressure map as input.

This section briefly describes the fundamentals of PA imaging. Further details can be found in [59], [60], and [61]. Fig. 2 illustrates PA signal generation and acquisition through two distinct processes. The first, known as the optical forward problem, determines the initial pressure generated by chromophores within the medium. Each endogenous or exogenous chromophore possesses a unique absorption coefficient at a specific light wavelength λ [62]

(1) $$\mu_{a}\left(r,\lambda \right)=\sum_{l=1}^{L}\alpha_{l}\left(\lambda \right)C_{l}\left(r\right)$$

where L denotes the number of chromophore types and $C_{l}$ and $\alpha_{l}(\lambda )$ denote the concentration and unit optical absorption spectrum of the lth chromophore, respectively.

The ultimate goal of PA imaging is to reconstruct the concentration of chromophores $C_{l}(r)$ at each position r using the known spectrum $\alpha_{l}(\lambda )$. For example, a primary target for many PAUS applications is the local blood concentration and its oxygenation level, which can be reconstructed from the PA-reconstructed optical absorption $\mu_{a}(r,\lambda )$ estimated at a number of wavelengths. The concentration of each chromophore contributes to the medium’s optical absorption coefficient at any specific wavelength. At the same time, most biological tissues are highly light scattering (or turbid), and light scattering $\mu_{s}(r,\lambda )$ also a function of both location and wavelength) is many times larger than optical absorption. The combination of optical absorption and scattering within the medium defines the optical fluence distribution $\Phi \left(r,\mu_{a},\mu_{s}\right)$, thereby determining the distribution of absorbed energy

(2) $$H\left(r,\mu_{a}(r,\lambda ),\mu_{s}(r,\lambda )\right)=\mu_{a}(r,\lambda )\Phi \left(r,\mu_{a}(r,\lambda ),\mu_{s}(r,\lambda )\right)$$

and, subsequently, the pressure excitation through thermalization

(3) $$p_{0}(r,\lambda )\;=\Gamma (r)\mu_{a}(r,\lambda )\Phi \left(r,\mu_{a}(r,\lambda ),\mu_{s}(r,\lambda )\right)\;\;=\Gamma (r)H\left(r,\;\mu_{a}(r,\lambda ),\mu_{s}(r,\lambda )\right)$$

where $\Gamma =\beta c^{2}/C_{p}$ is the Gruneisen coefficient, c is the sound speed, β is the coefficient of volumetric thermal expansion, and $C_{p}$ is the specific heat at constant pressure, which, in general, are all functions of r. The second process, referred to as the acoustic forward model, determines the US signals acquired by the imaging system arising from the initial pressure. PA data are influenced by both the acoustic properties of the medium and the characteristics of the detector(s).

To quantify the volumetric distribution of chromophore concentration, the overall inverse problem must be solved. First, to determine the initial pressure distribution from recorded data, the acoustic inverse problem must be addressed. This process, and the resultant map of initial pressure, are commonly referred to as “PA reconstruction” and the “PA image,” respectively.

The subsequent step estimates chromophore concentrations using PA images and volumetric maps of Γ(r) and Φ(r,λ). Multiple optical wavelengths are often used to improve these estimates since each chromophore has a unique optical absorption spectrum. This approach is commonly called “PA spectroscopic imaging” or “PA quantitative imaging.” It is not trivial and requires separate analysis. Details on optical fluence reconstruction methods are summarized in [61], [63], and [64].

The simple sensor geometry used in many PAUS systems is determined by the physical access available to US probes for a specific medical application. The limited size and bandwidth of these probes affects the quality of reconstructed PA images, often greatly misrepresenting the shape of volumetric chromophore distributions (endogenous or exogeneous). For example, large blood vessels and microvessel networks containing strongly absorbing blood can be greatly distorted. In Sections II-A–II-C, these technical difficulties will be described in detail. Thus, this article primarily focuses on reconstructing the volumetric shape of absorbers, an essential component of complete PA inversion.

### A. Photoacoustic Signal

The spatio-temporal pressure p(t,r) at time t after initial pressure generation is given by the PA equation [59]

(4) $$\left(\nabla^{2}-\frac{1}{c^{2}}\frac{\partial }{\partial t}\right)p(t,r)=-\frac{\beta }{C_{p}}\frac{\partial H(t,\;r)}{\partial t}.$$

If the excitation laser pulse is short enough to satisfy stress and thermal confinement conditions, it can be approximated as an infinitesimally short pulse, δ(t) and hence the H(t,r) can be represented as H(t,r)=H(r)δ(t). Then, the temporal profile of pressure at the position of an acoustic detector, $r^{\prime }$, can be expressed as a Rayleigh integral over the distribution of heat release [24]

(5) $$p\left(t,r^{\prime }\right)=\frac{\Gamma }{4\pi c^{2}}\frac{\partial }{\partial t}\left[\int \;\frac{dr}{\left|r\;-\;r^{\prime }\right|}H(r)\delta \left(t-\frac{\left|r\;-\;r^{\prime }\right|}{c}\right)\right].$$

Assume a transducer contains J detection elements. Then, the signal recorded by the jth element can be represented as

(6) $$y\left(t,r_{j}^{\prime }\right)=\psi \left(p\left(t,r_{j}^{\prime }\right)\right)+n\left(t,r_{j}^{\prime }\right)$$

where ψ(⋅) and $n(t,r_{j}^{\prime })$ denote the system function and acquisition noise, respectively. The goal of PA image reconstruction is to map initial PA pressure $p_{0}(r)$ [or the function H(r)] from the measurements $\left\{y(t,r_{j}^{\prime })|j=\;1,\ldots ,J\right\}$.

### B. Detection Element Geometry

**Fig. 3.**
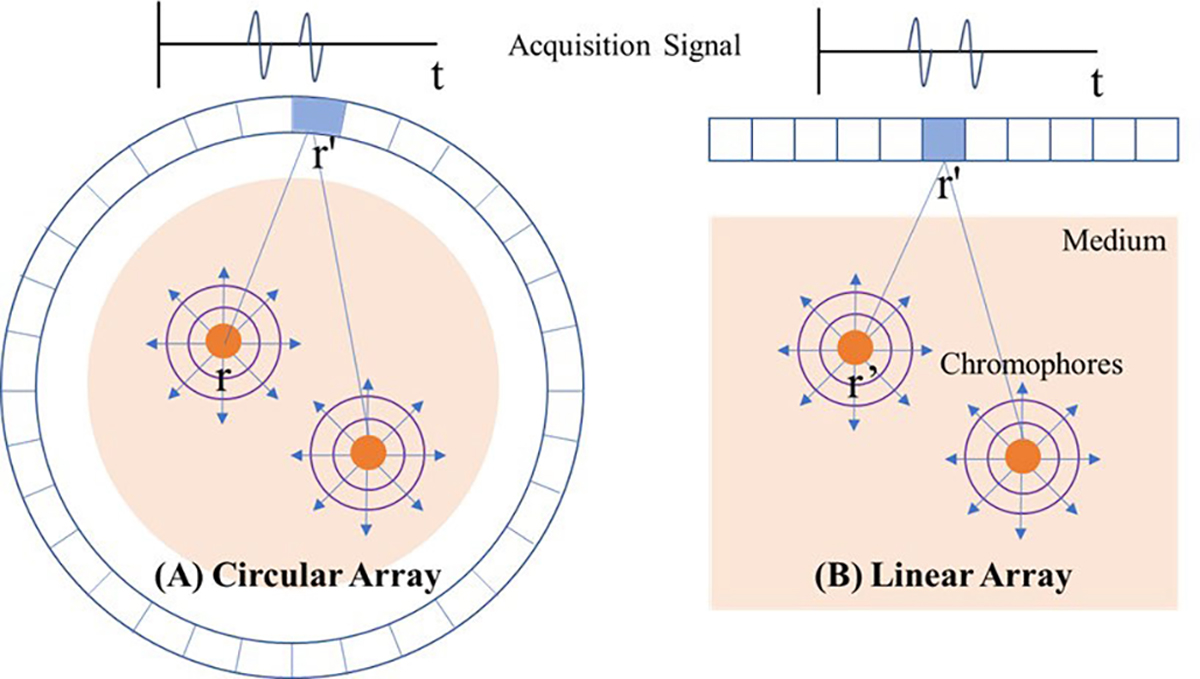
PA signal generation and detection for a simple 2-D example. Chromophores located at different r in the medium generate PA waves at t=0, and sensors located at $r^{\prime }$ receive them at $t=\left(r-r^{\prime }\right)/c$, where c denotes their propagation speed. (a) Circular sensor array surrounds the medium. (b) Linear array is at the top of the medium. The signals received from every sensor are N-shaped if the chromophores are circular with diameter D, where D determines the duration of the N-shape. This assumes that light attenuation within the chromophore sphere can be neglected. If the sensor receives only over a limited frequency range, the signals are bandwidth-limited N-shaped.

An ideal PACT system must have a cylindrical or spherical geometry for the transducer sensor array surrounding the measurement volume to detect all PA signals originating from every chromophore in the volume [25], [65]. For 2-D sectional imaging, therefore, the object must be enclosed by a circular array as shown in Fig. 3(a). The spatial image resolution is determined by the frequency response of a single sensor in the array assuming that the detectors are point-like. If the detectors are not point-like, then their specific geometry must be taken into account. For instance, a focused array is usually employed to realize high elevational resolution.

Artifacts are likely present in 2-D imaging because each sensor can inevitably receive signals from outside the imaging plane since light is diffused over three dimensions. To illustrate, consider a scenario where a strong point absorber lies outside the plane of the detector but is close to the origin of the circular array. Even if the array has a tight elevational focus, acoustic waves generated by this absorber will be detected by all array elements. Since the arrival time of these signals does not coincide with the in-plane propagation time from the center of the ring to a given detector, any 2-D reconstruction cannot eliminate this signal, resulting in a “blob” artifact rather than a well-defined point at the image center. Although these artifacts can be significant, we will limit the scope of this article to 2-D reconstructions neglecting out-of-plane artifacts. Future studies must address full 3-D reconstructions to ensure robust PA imaging under all conditions.

The typical PAUS platform does not even approach an ideal 2-D geometry because a standard clinical transducer, typically a linear sensor array as shown in Fig. 3(b), has a greatly limited view, i.e., PA signals are recorded within an aperture much less than 180°. This geometry can be easily manipulated and brings PAUS imaging to a wide range of medical applications where US is currently used. However, it creates an ill-posed condition that degrades absorber shapes in the reconstruction process. Thus, PA signals recorded under limited view conditions impose severe shape artifacts even for simple objects. The condition is exacerbated if the target is both discrete and not small compared to an acoustic wavelength at the central operating frequency of the array.

**Fig. 4.**
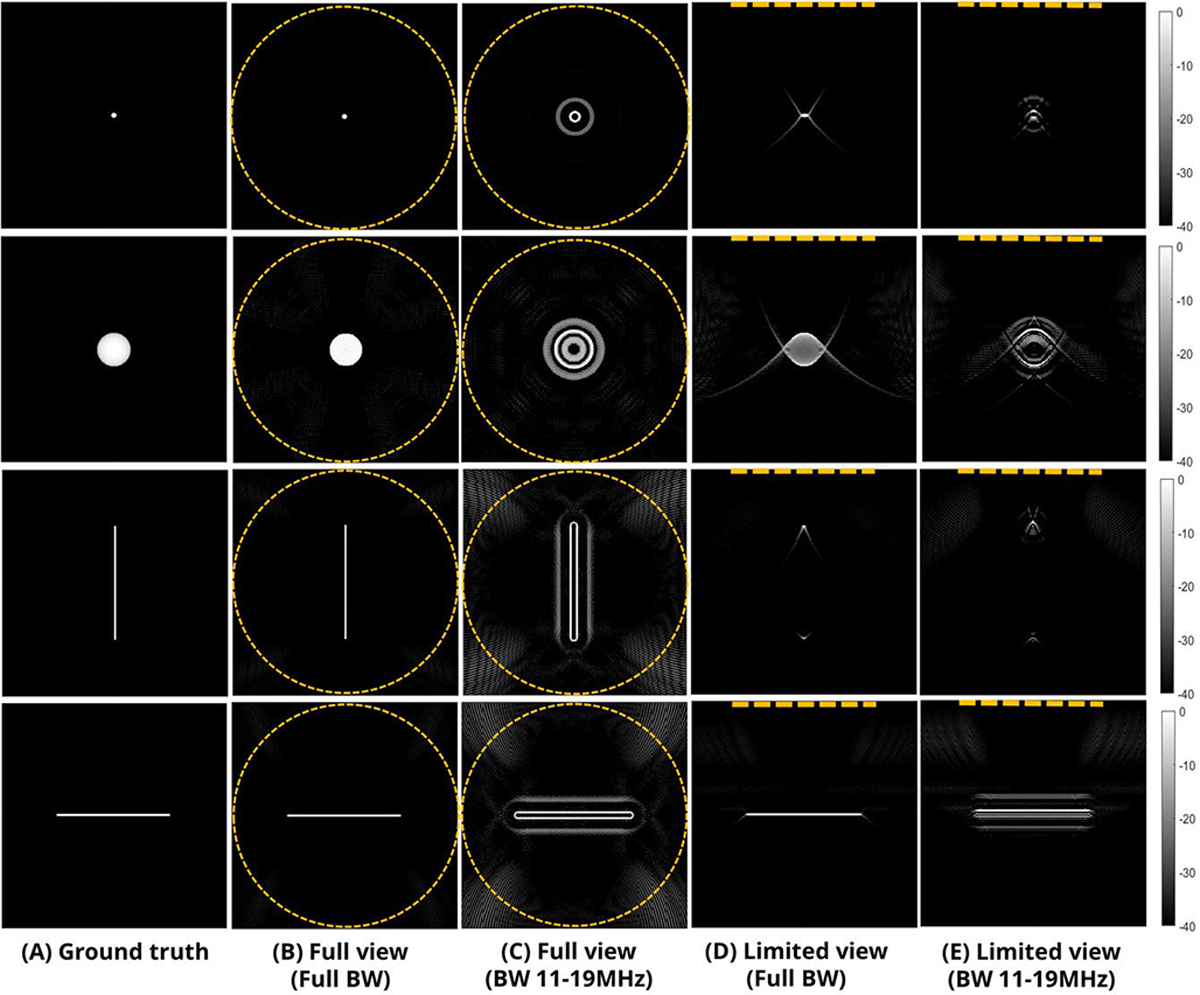
Simulation results using standard filtered back-projection reconstruction. (a) Four example object shapes. (b)–(e) Reconstructions when the acquisition conditions are (b) circular array with full bandwidth, (c) circular array with limited bandwidth (11–19 MHz), (d) linear array with full bandwidth, and (e) linear array with limited bandwidth (11–19 MHz). Array geometry illustrated with dashed orange line for each case and all images are shown on a log-scale colormap (40-dB range).

These points were thoroughly examined in simulation. Fig. 4 displays reconstructions using a standard method (introduced in Section II-C) under various acquisition conditions. To simplify these simulations, light and US attenuations were omitted, and postimage processing steps were skipped to focus solely on visualizing the pattern changes caused by ill-posed conditions. The details are summarized in the Appendix. When the geometry used a circular array with full bandwidth, accurate reconstruction was achieved, as shown in Fig. 4(b). However, narrowing the bandwidth during acquisition preserved the object shapes but introduced ripple artifacts, as demonstrated in Fig. 4(c). On the other hand, when the acquisition view was limited, the shapes became distorted, as illustrated in Fig. 4(d)–(e).

Image artifacts are exacerbated if the target was not small compared to an acoustic wavelength at the central operating frequency of the array. If the absorption field $\mu_{a}(r)$ at a specific optical wavelength slowly varies around position $r_{0}$, the emitted signal from $r_{0}$ is extremely weak because of the derivative term with respect to time in (5). This signal was even weaker if the sensor has limited bandwidth. For example, as shown in Fig. 3, a circular absorber generates a bandwidth-limited N-shaped signal. If the diameter of the absorber is large, it causes relatively strong signals at boundaries but weak signals around the center. Only a full aperture and wide signal bandwidth can recover the low frequencies required for a faithful reconstruction of a circle. Thus, only targets limited to strong, sparse absorbers whose shape is point-like or finely vascular, protruding from other weak absorbers regarded as background in a medium, can lessen the ill-posedness of this geometry. In the frequency domain, the signal components for this class of absorber are distributed evenly across the total domain, or dominantly in the high-frequency domain. As a result, even though the derivative term and limited bandwidth may significantly weaken low-frequency components, PAUS image reconstruction is still tractable. However, one exception is the vertical vascular shape, as shown in Fig. 4, because the array sensors cannot receive plane waves propagating horizontally. This effect is explained in the frequency domain in [37]. Simulations in [66], [67], and [68] have also revealed similar artifacts for this detection geometry.

### C. Conventional Image Reconstructions Schemes

Many papers proposed analytical approaches to map the initial pressure $p_{0}(r)$ [or heating function H(r)] from PA measurements $y\left(t,r^{\prime }\right)$ given a well-posed condition. When the acquisition view and detector bandwidth are full, the detector function f(⋅) is linear and the noise $n\left(t,r^{\prime }\right)$ is zero, the simplified UBP method [25], [69] can be expressed as

(7) $$\tilde{p}(r)=-\varrho \int \;\frac{\partial y\left(t,\;r^{\prime }\right)}{\partial t}\delta \left(t-\frac{\left|r\;-\;r^{\prime }\right|}{c}\right)dr^{\prime }$$

where the constant ϱ depends on the transducer geometry. If the density of detector elements is above the spatial Nyquist sampling rate, the discrete version of UBP can reconstruct PA sources perfectly from measurements $\left\{y(t,r_{j}^{\prime })|j=1,\ldots ,J\right\}$ as

(8) $$\tilde{p}\left(r\right)=-{\varrho \sum_{j}\frac{\partial y\left(t,\;r_{j}^{\prime }\right)}{\partial t}|}_{t=\left|r-r_{j}^{\prime }\right|/v_{s}}.$$

The UBP method can also be used when the view and bandwidth are limited. As shown in Section II-B, the main target should be small or vessel-like. Since the strong signals from compact absorption sites against a uniform background are short pulses, the derivative term can be ignored in (8). Instead, postprocessing to smooth the wave oscillation can be used as

(9) $$\tilde{p}\left(r\right)=F\left({\sum_{j}y\left(t,r_{j}^{\prime }\right)|}_{t=\left|r-r_{j}^{\prime }\right|/c}\right)=F\left(\sum_{j}f\left(r,j\right)\right)$$

where F(⋅) denotes a processing operator, such as the Hilbert transform, and $f(r,j)=y(|r-r_{j}^{\prime }|/c,r_{j}^{\prime })$. This approach is very similar to delay and sum (DAS) beamforming used in radar applications or clinical US imaging [70]. As shown in (9), before summing, a delay $t=|r-r_{j}^{\prime }|/c$ is applied to account for the variable propagation distance/time from the source at position r to the sensor at position $r_{j}^{\prime }$.

Another reconstruction approach uses time reversal (TR) to solve the wave inversion equation by simulating a wave back-propagating to the image field from each sensor [40]. TR utilizes time-reversed reemission of received signals to focus the energy at the desired imaging location. By iteratively computing the wave field, TR can account for aberrations if medium heterogeneities are not high, but the computational burden associated with the iteration process is a practical limitation. If tissue is not fully enclosed by detectors, the resulting image quality is compromised because TR uses the DAS framework.

Equations (3), (5), and (6) can be simply expressed as $y=Ap_{0}$ where A denotes the forward operator generating measurement y from source $p_{0}$. Both DAS and TR methods cannot invert this operation uniquely due to limited view and bandwidth conditions. Some groups [71], [72] adopted penalties (regularizers) based on prior knowledge to obtain a more plausible solution, where optimization takes the form

(10) $${\tilde{p}}_{0}=\underset{p_{0}}{argmin}{∥y-Ap_{0}∥}_{2}^{2}+g\left(p_{0}\right)$$

and $g\left(p_{0}\right)$ denotes the penalty term. However, it is challenging to identify a penalty function that is general enough for all samples. In most cases, there is no closed-form solution available. Iterative algorithms approaching real-time rates are possible for simple objects and PA data from high SNR, broad bandwidth, and near-full view tomographic detection [73], [74], or for aberration correction induced by variance in US speed [75]. However, iterative inversion methods have not yet been proven or experimentally demonstrated to converge to the actual volumetric distribution of heat release for very sparse PA data (very limited bandwidth and view, and typically low SNR) acquired from a conventional PAUS geometry [76], [77].

## III. Deep Learning for Imaging

### A. Supervised Learning

DL is part of a broader family of machine learning methods based on artificial neural networks. The fundamental learning technique fits large sets of training data using the model to find features (patterns) to adapt properly to new data [78]. Given each data sample as an input, the model outputs the scalar, vector, matrix, or higher dimensional tensor type, depending on the imaging task. Supervised learning [79] takes advantage of an instructor concept to optimize model parameters that minimize the cost (loss) function measuring the discrepancy between ground truth (answer) and model output. During training (learning), the model decomposes data into shape, texture, or abstract features to facilitate the recovery of intended visual data.

The learning process can be expressed as

(11) $$\hat{\theta }=\text{arg}\;\text{min}\sum_{j=1}^{J}l\left(x^{\left(j\right)},g\left(y^{\left(j\right)};\theta \right)\right)$$

where J denotes the number of training samples, l(⋅) denotes the loss function, $x^{\left(j\right)}$ denotes the jth ground-truth, and ${\overset{ˆ}{x}}^{\left(j\right)}=g\left(y^{\left(j\right)};\theta \right)$ denotes the predicted output of the DL model g with the parameter set θ when the input is the jth data sample $y^{\left(j\right)}$. To find the optimal set $\overset{ˆ}{\theta }$, each parameter is gradually updated by gradient descent approaches. The stochastic gradient descent framework using batches can leverage GPU parallel computing to train large-scale neural networks efficiently.

As described in [78], this approach can be viewed as minimizing the Kullback–Leibler (KL) divergence $D_{KL}\left({\overset{‾}{p}}_{data}(x)∥p_{\text{model}}(x|y;\theta )\right)$ where $p_{\text{model}}(x|y;\theta )$ denotes the probability distribution over data space x given by the input y and parameter set θ, and ${\overset{‾}{p}}_{data}(x)$ denotes the empirical distribution defined by the training data. In this context, the optimization process aims to align the model distribution with the empirical distribution, ideally representing the true data-generating distribution $p_{\text{data}}(x)$. Using Gaussians for the distributions minimizes mean squared error (mse) as the loss function l(⋅).

### B. Deep Learning Model

**Fig. 5.**
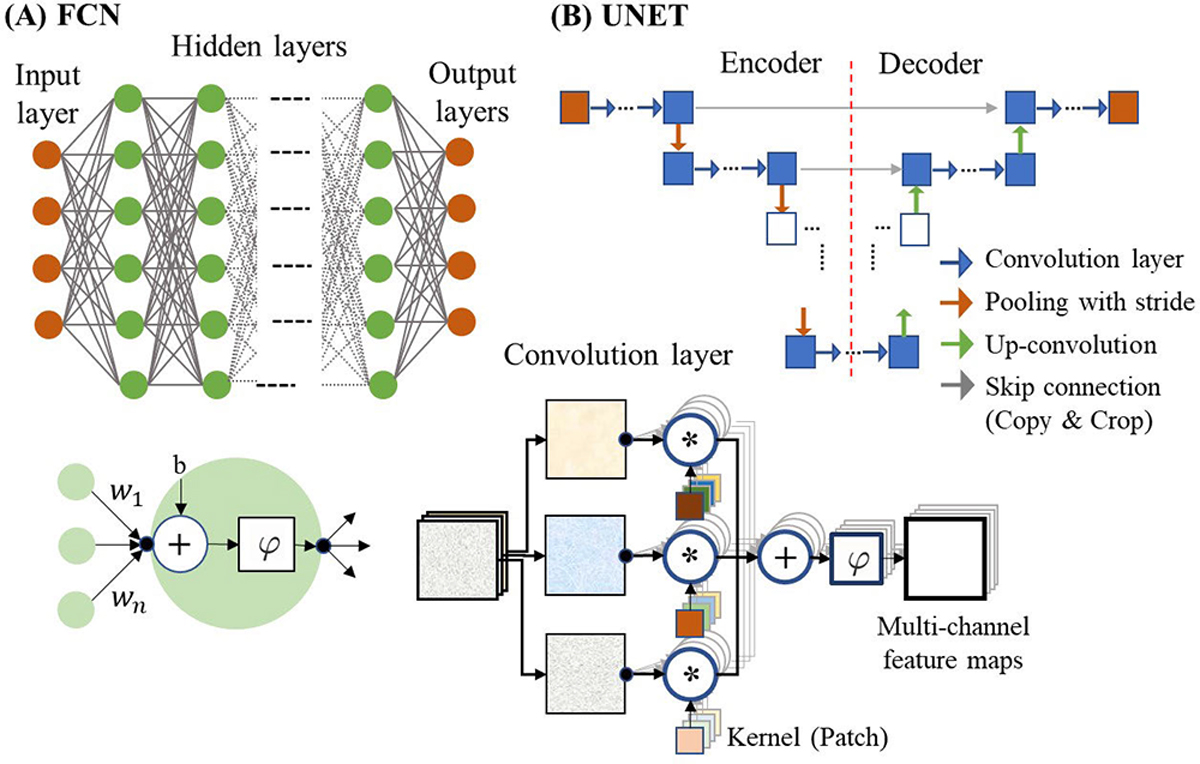
(a) FCN. The filled circle represents a neuron. One neuron takes values from neurons in the previous layer, linearly combines the numbers, performs the nonlinear operation, and passes the resultant number into the neurons in the next layer. (b) UNET. This network leverages a CNN. Each arrow indicates an operation. The pivotal layer is the convolutional one illustrated below. Inputs and outputs to this layer are multichannel maps. Input maps are convolved with small kernels (patches/filters) and the resultant maps are summed. The resultant map passes through the nonlinear function to generate one output map. The same procedure is performed for other maps.

Here, we briefly introduce DL models that are best suited to medical imaging. A fully connected network (FCN) is the basic DL model, as shown in Fig. 5(a), where it contains an input layer, hidden layers, and an output layer. Every neuron (perceptron) in a hidden layer is connected to the neurons in the previous and next layers and sums all inputs, applying a nonlinear operation (activation) to the resultant as

(12) $$h^{\left(l+1\right)}=\varphi (b^{\left(l\right)}+W^{\left(l\right)}h^{\left(l\right)})$$

where $h^{\left(l\right)}$ denotes the output of neurons in the lth layer or the input of neurons in the l+1th layer, $W^{\left(l\right)}$ denotes the weights, $b^{\left(t\right)}$ denotes biases, φ(⋅) denotes the activation function, such as a rectified linear unit (Relu), and $h^{\left(l+1\right)}$ denotes the output of neurons in the l+1th layer. For a regression task, the output layer has no special activation function. It has been shown that the hierarchical model excels at capturing complex nonlinear relationships in data and extracting abstract features relevant across different instances of a problem [78].

Convolutional neural networks (CNNs) have performed well for various imaging tasks because they leverage common statistical properties of images such as local invariance [80], [81]. The basic network block is the convolutional layer illustrated in Fig. 5(b). Assume one image is input to the layer; as each small filter travels over the entire image, it can highlight the specific pattern in a local area and store the degree in the feature map. In the next layer, the feature maps are convolved with new filter banks to extract deeper features and store the results in the new feature maps [see Fig. 5(b)]. This process is repeated for the next layers as

(13) $$D_{j}^{\left(l+1\right)}=\varphi \left(\sum_{k=1}^{K^{\left(l\right)}}D_{k}^{\left(l\right)}\;\text{*}\;p_{kj}^{\left(l\right)}+b_{j}^{\left(l\right)}\right)$$

where the operation * denotes convolution, $K^{\left(l\right)}$ denotes the number of feature maps (channels) in the lth layer, $D_{k}^{\left(l\right)}$ denotes the kth feature map (channel) in the lth layer, $p_{kj}^{\left(l\right)}$ denotes the kth filter (patch or kernel) in the lth layer generating the jth feature map $D_{j}^{\left(l+1\right)}$ in the next layer, $b_{j}^{\left(l\right)}$ denotes the bias, and φ(⋅) denotes the activation function. Convolutional layers can reduce computational complexity due to parameter sharing and spatial localization properties. They have a significantly lower number of connections (trainable parameters) compared to fully connected layers, and are suitable for large-scale datasets or resource-limited scenarios.

UNET [82] is one of the CNN networks well-suited to image-to-image mapping. As shown in Fig. 5(b), the structure consists of: 1) the encoder conducting multiscale image decomposition using convolutional layers and downsampling operators and 2) the decoder recovering an image from multiscale feature maps using convolutional layers and upsampling operators. The concept is similar to discrete wavelet decomposition and reconstruction using filter banks to identify multiresolution features [83]. The “skip connection” concatenates feature maps in the decoder with those in the encoder, so that the decoder can access not only deep features but also low-level features. Currently, UNET has been modified by adding attention modules in the convolutional layers or replacing skip connections into them [84]. Attention focuses the model on key feature maps and suppresses redundant features [85], [86]. For example, channel attention and spatial attention assign weights to different channels and spatial locations based on their importance for the task, respectively.

**Fig. 6.**
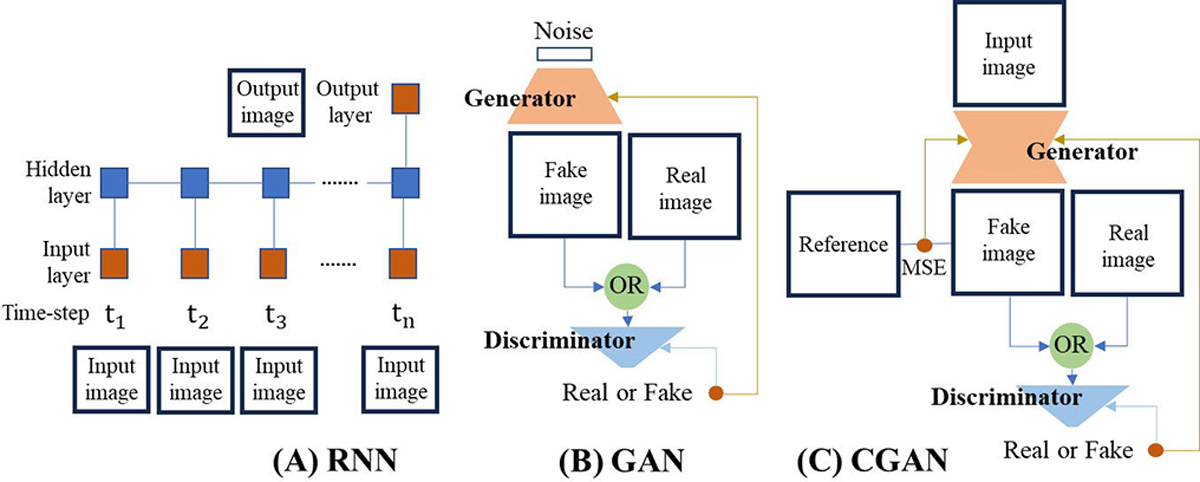
(a) RNN. At every time-step, an image is input. In the hidden layer, the hidden units are connected to convey information to the next or previous time-step. Trainable parameters are shared in every time-step. The output image can be produced at any time-step. (b) GAN. The generator creates a fake image to deceive the discriminator. The discriminator is a classifier to distinguish between real and fake images. (c) CGAN. The generator generates a fake image given the condition (input image). It attempts to make the image as close as the given reference as well as deceive the discriminator.

The hybrid architecture combining a CNN with a recurrent neural network (RNN) has been developed for multiframe images [87], [88]. As shown in Fig. 6(a), the RNN structure is specialized to sequential data by inputting data at every time-step. The network has recurrent connections between hidden units, simply expressed as

(14) $$h^{\left(t\right)}=g(h^{\left(t-1\right)},x^{\left(t\right)};\theta )$$

where t denotes the time-step, h denotes the hidden unit, x denotes the input unit, and g(⋅;θ) denotes the sharing neural network with trainable parameters θ over t. The network can produce an output at every time-step or at specific time-step (in general, the last step). Like CNN, RNN can reduce complexity due to parameter sharing and localization across time-steps.

Generative adversarial networks (GANs) [89], [90] can output more plausible images using ingenious cost (loss) functions for optimization beyond standard metrics such as mse or mean absolute error (MAE). The GAN contains two neural networks: a generator and a discriminator. The generator captures the real image distribution and creates a realistic fake image while the discriminator discriminates fake from real samples. As shown in Fig. 6(b), the generator G maps from random (noise) space $z∼p_{z}(z)$ to image space $x∼p_{g}(x)$, and the discriminator takes either the generated image or real image to output the probability that the image came from real samples rather than fake samples. GAN updates the parameters in the two networks using the minmax optimization problem as

(15) $$\underset{G}{min}\;\underset{D}{max}\;V(D,G)=E_{x∼P_{\text{data}}(x)}[logD(x)]\;\;+E_{z∼p_{z}(z)}[log(1-D(G(z))]$$

where $P_{data}(x)$ denotes the distribution of real image samples. For instance, if the input is real, the discriminator attempts to output the number closest to 1 by maximizing the cost. This architecture can be used for data augmentation in medical imaging [91], [92].

When the task is image enhancement or reconstruction from low-quality image or raw data, the conditional GAN (CGAN) [93] has been adopted, as shown in Fig. 6(c). Pix2pix [94] is one of the best-known CGANs for image-to-image translations. In this architecture, the noise vector is replaced by the image or data as a condition, and the generator is trained to create the image close to a reference by minimizing the combination of the KL-based GAN cost and mse or MAE. Using only mse/MAE, a blurry image is often produced [95]. The addition of GAN cost, however, helps extract details in the reference and create a more sophisticated image by attempting to deceive the discriminator.

### C. Deep Learning Frameworks for PA Image Reconstruction

**Fig. 7.**
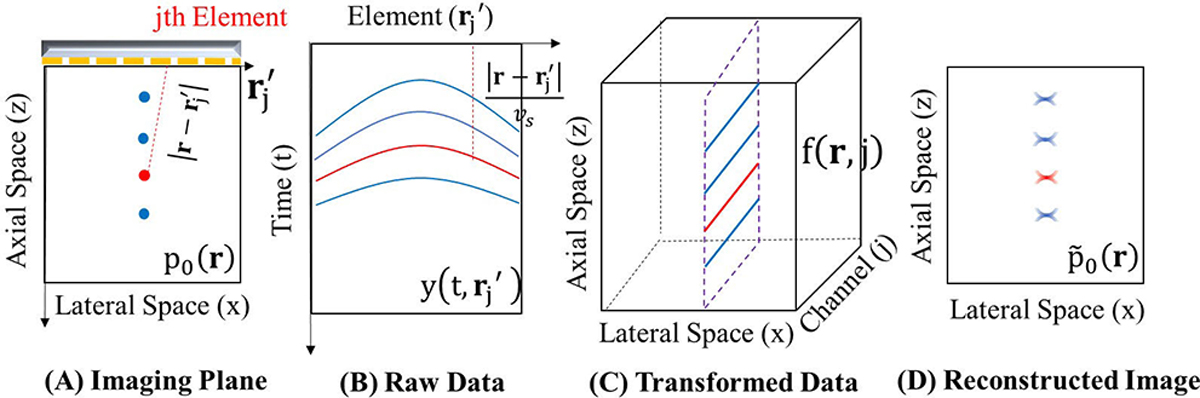
(a) Imaging plane and sensors (linear array). The example presented here assumes four strong point-like chromophores. (b) Channel (sensor) data (raw data). Four wavefronts are shown in the data domain. (c) Transformed channel data. Data samples corresponding to the time of flight from each position r are aligned along the channel axis. (d) Standard DAS imaging result (reconstructed image). All figures are reproduced with permission from [37].

Several studies have explored DL frameworks for the PAUS geometry. The selection of papers for this review aimed to highlight recent discoveries concerning learning structure and/or experimental in vivo results in PA reconstruction within the context of the PAUS geometry. All PA imaging work presented here adopted supervised learning to overcome limited view and bandwidth problems. In every reconstruction task, the output was commonly a PA image (2-D matrix) mapping initial pressure $p_{0}$. However, the input to the DL model varied considerably. As shown in Fig. 7, input data can be categorized into three types: 1) sensor, or channel, data; 2) preprocessed (transformed) channel data; and 3) reconstructed images using a conventional method such as DAS or TR. Table I summarizes data acquisition conditions and proposed DL models for the work reviewed here.

Waibel et al. [27] proposed two distinct DL architectures. The first utilized a standard UNET framework with a rough DAS image as the input. The second model, derived from the UNET backbone, replaced each skip connection with a convolutional layer featuring a large kernel size and a large step size (called stride) at which the kernel moves across the data. This modification converted the high-sampled temporal domain in the encoder to the low-sampled spatial domain in the decoder. They used simulated (in silico) data from a linear transducer array obtained solely from circular targets that mimicked vessel cross sections.

**Fig. 8.**
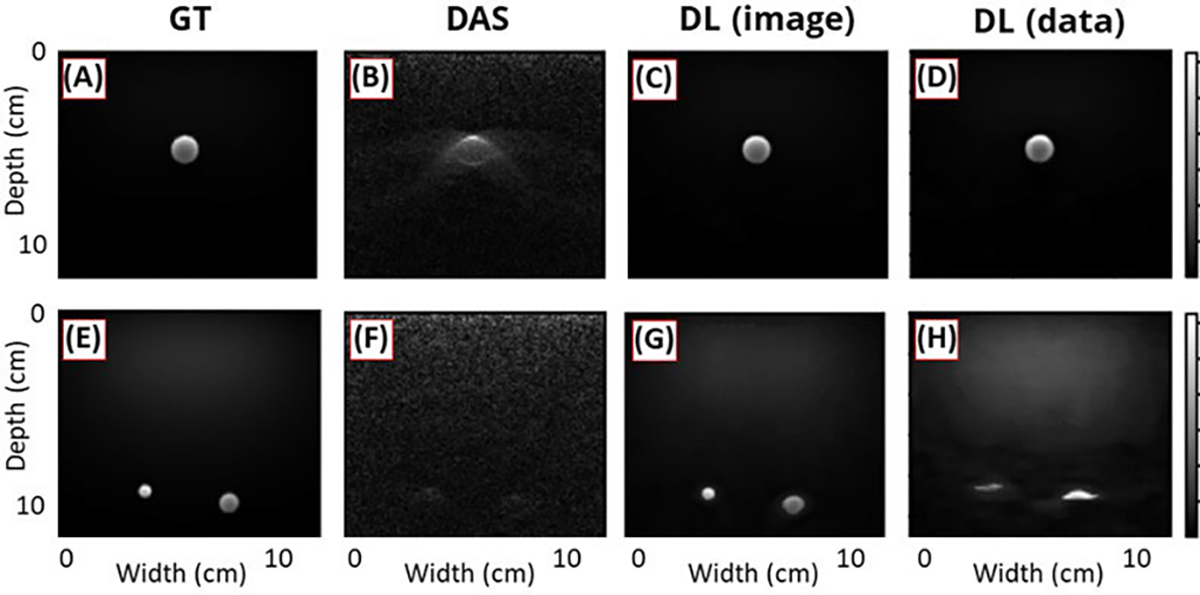
Simulation test results using (a)–(d) one circular target in the near field and (e) and (f) two circular targets in the far-field. (a) and (e) Ground truth. (b) and (f) DAS results. (c) and (g) DL results reconstructed from the DAS image. (d) and (h) DL results reconstructed from channel data directly. All figures are reproduced with permission from [27].

Fig. 8(a)–(d) demonstrated that both models reconstructed target shapes more accurately compared to standard methods. For targets located in the far-field [see Fig. 8(e)], the models predominantly restored the objects [see Fig. 8(g) and (h)], unlike DAS [see Fig. 8(f)]. Interestingly, despite extremely faint object traces in the DAS image, the first DL model, which was fed with the DAS image, restored the objects. The second model, which used channel data as input, had a larger number of trainable parameters than the first. However, it produced more distorted results compared to the first model. This suggests that the translation from data to image is considerably more challenging than image-to-image translation, necessitating more sophisticated structures specifically designed for this mapping. Although the parameters of the tissue-mimicking phantom used in the simulations may not perfectly represent real-world situations, this study is valuable as it represents one of the initial attempts to apply a DL approach to PA reconstruction and highlights the potential of DL in the field.

As observed in the literature, mapping channel data directly to an image is challenging, even though channel data contain more physical information about the target and acquisition conditions. Although the drawbacks of mapping image-to-image are rarely discussed in the literature, it can lead to artifacts and low generalization, especially when dealing with complex targets due to limited information. Lan et al. [96] developed a UNET-based model called YNET, which addresses these challenges by simultaneously feeding both channel data and a DAS image into the network. The model consisted of two encoder modules inputting both channel data and the image, and one decoder module that produced the final image. The key concept behind this approach was that the two encoders shared their feature maps with the decoder using skip connections at every scale.

**Fig. 9.**
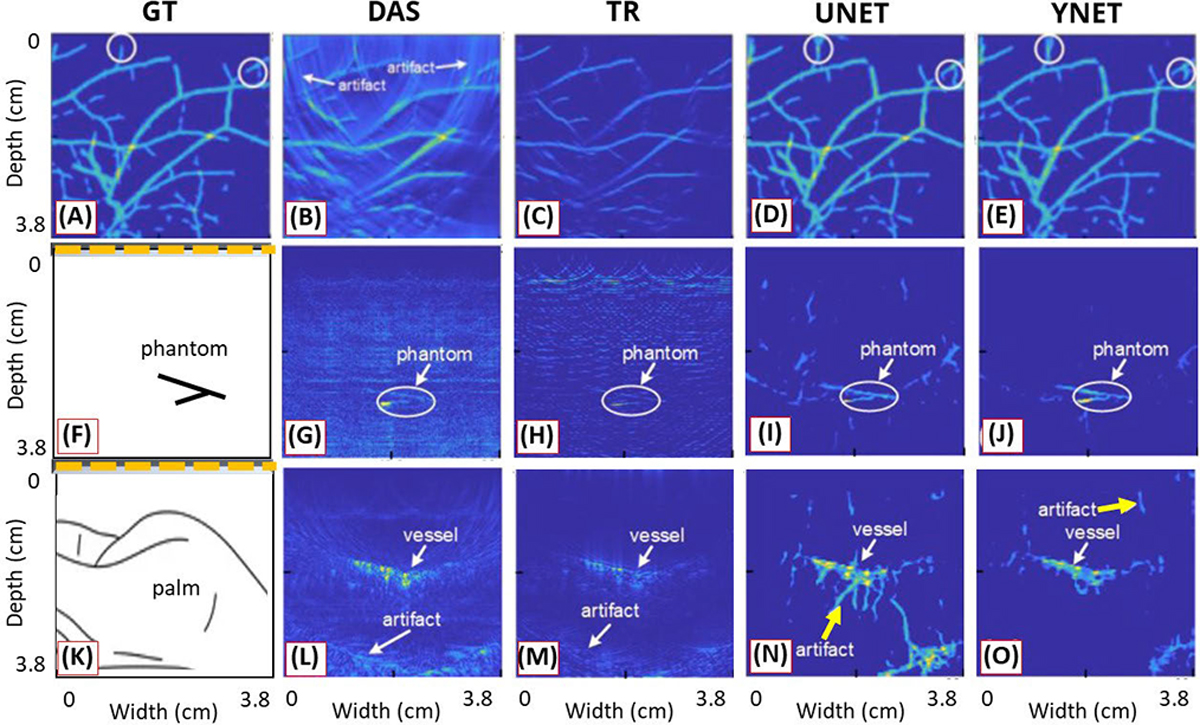
Reconstruction results using (a)–(e) synthetic vascular data, (f)–(j) in vitro phantom data acquired from chicken breast tissue with two pencil leads inserted, and (k)–(o) in vivo data acquired from a human palm. (a), (f), and (k) Ground-truth or acquisition field illustrations (b), (g), and (l) DAS results. (c), (h), and (m) TR results. (d), (i), and (n) Results for UNET fed by DAS images. (e), (j), and (o) Results for the proposed DL (YNET) fed by both channel data and the DAS image. All figures are reproduced with permission from [96].

Compared to a method employing two independent networks, the shared decoder in YNET could leverage features from both channel data and image domains, while also reducing the number of trainable parameters. Channel data were acquired using a 7-MHz linear transducer with 80% bandwidth. For target objects in simulations, vascular structures were extracted from fundus oculi images [101], and training data were synthesized under limited view and bandwidth conditions. The DL model was trained with synthetic data and the model was tested using simulation data, in vitro chicken breast data, and in vivo human palm data, demonstrating that the proposed model significantly improved imaging performance, as shown in Fig. 9. The method presented targets with higher contrast compared to standard methods (DAS and TR) and fewer artifacts than a UNET model fed by only a DAS image.

Kim et al. [37] proposed a new form of input data for a UNET model. As shown in (9), channel data y can be transformed to f based on the time of flight of an US pulse from a potential PA source. Fig. 7(c) illustrates this conversion when the imaging plane is [r=(z,x)]. Discretization of f(r,j) creates multichannel 2-D matrices (3-D tensor). Specifically, r is sampled using image pixel positions and assigned channel data samples based on the time-of-flight $\left(t=|r-r_{j}^{\prime }|/v_{s}\right)$ from each pixel position to each sensor (channel). Using this multichannel data as input, the DL model can more effectively access the primary data samples contributing to each pixel position. If the pixel resolution is sufficiently high, the model can handle raw data with minimal information loss in both the encoder and decoder.

**Fig. 10.**
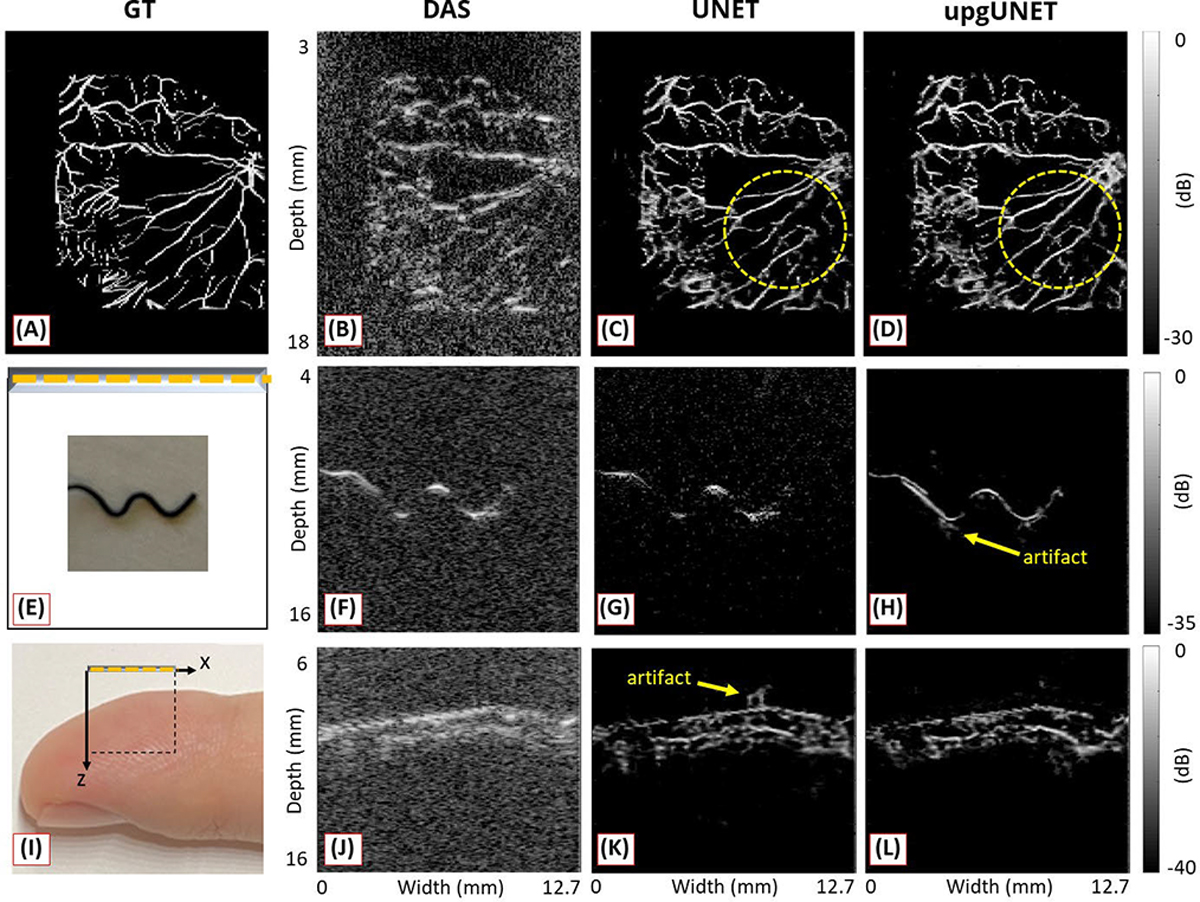
Reconstruction results using (a)–(d) synthetic vascular data, (e)–(h) in vitro phantom data acquired from a “W” shape wire, and (i)–(l) in vivo data acquired from a human finger. (a), (e), and (i) Ground-truth or acquisition field illustrations. (b), (f), and (j) DAS. (c), (g), and (k) Results of UNET fed by DAS images. (d), (h), and (l) Results of UNET fed by transformed channel data. All figures are reproduced with permission from [37].

The target of this study was vascular structures, and thus a fundus oculi database [101] was also employed as a reference. During simulation, real acquisition conditions were mimicked (linear probe, center frequency: 15 MHz, 3-dB bandwidth: 8 MHz), and synthetic raw data were generated to train the model. Results showed the effectiveness of feeding preprocessed data using synthetic vascular data, in vitro data (w-shape wire), and in vivo data (human finger). This approach restored more detailed structures with fewer artifacts compared to inputting the DAS image, as illustrated in Fig. 10.

Vu et al. [97] introduced a GAN-based model to enhance images acquired with the PAUS geometry. While the traditional GAN loss function [see (15)] is typically based on KL or Jensen–Shannon (JS) divergence [89], these approaches often fail because of gradient vanishing and mode collapse [102], [103]. The metric turns infinite when the generated distribution does not overlap with the real distribution. Instead, these researchers adopted Wasserstein GAN (WGAN) [102], which utilizes the continuous loss called the Wasserstein distance (also known as Earth’s Mover distance) to enhance stability (convergence) during min-max optimization. They employed a CGAN framework and constructed a loss function that combines GAN loss with mse.

**Fig. 11.**
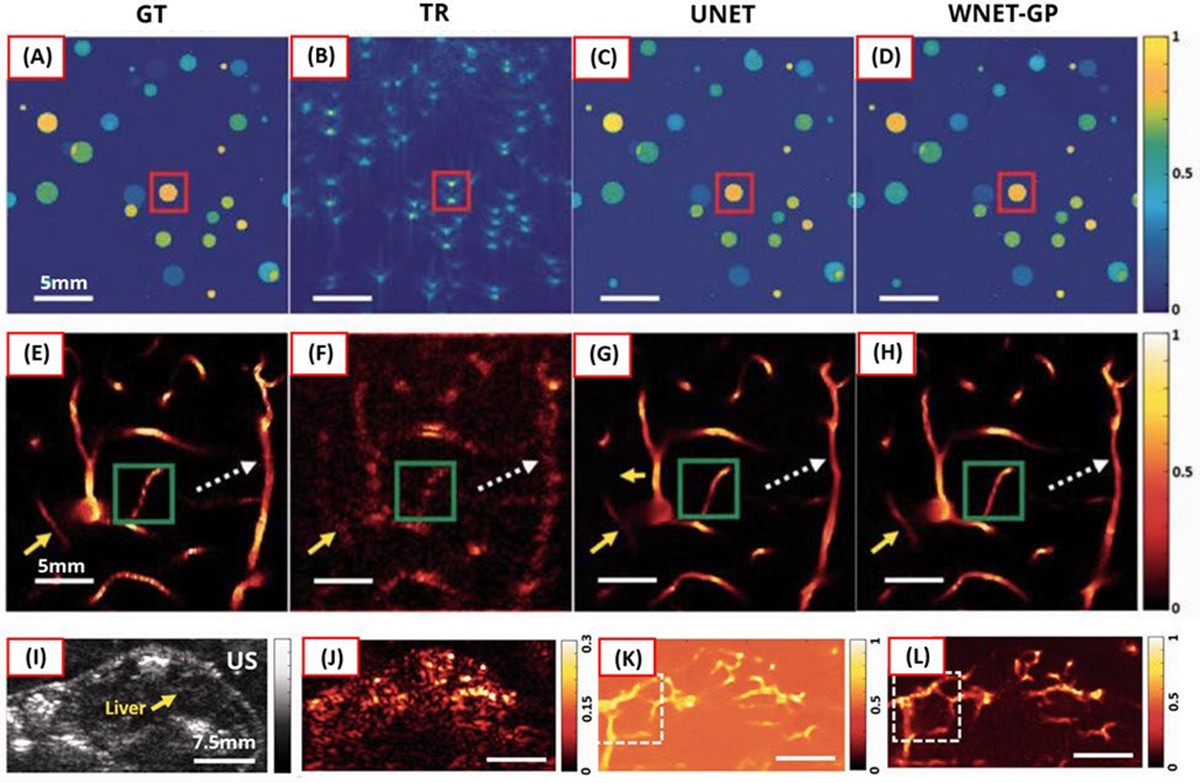
Reconstruction results using (a)–(d) synthetic circular disks data, (e)–(h) synthetic vascular data, and (i)–(l) in vivo data acquired from the mouse trunk. (a) and (e) Ground-truth. (i) US B-mode image. (b), (f), and (j) TR results. (c), (g), and (k) Results of UNET fed by TR images. (d), (h), and (l) Results of the proposed DL (WNET-GP) fed by TR images. All figures are reproduced with permission from [97].

As described in Section III-B, GAN loss guides the generator to produce image samples aligned well with the distribution of real image samples, thus deceiving the discriminator. The generator in their model used an initial TR image. For reference images, they generated simulated images with randomly distributed circular disks, and they also employed a brain vascular database obtained through two-photon microscopy [104]. Training and testing data were generated in a simulation environment assuming a linear transducer with a center frequency of 5 MHz and a 3-dB bandwidth of 60%. Additionally, for in vivo testing, they imaged skin vasculature in the trunk of a mouse. As demonstrated in Fig. 11, the proposed model improved the visibility of target structures, including vertical vessels. Compared to a standard UNET model, the proposed model preserved fine structural details with higher contrast.

An LED has some advantages as a light source in PAUS systems. It is cost effective, can operate at very high repetition rates, and can switch between different optical wavelengths quickly. However, its low fluence produces very weak PA signals. In their study, Hariri et al. [98] developed a DL framework specifically designed to enhance image contrast in LED-based PAUS systems.

**Fig. 12.**
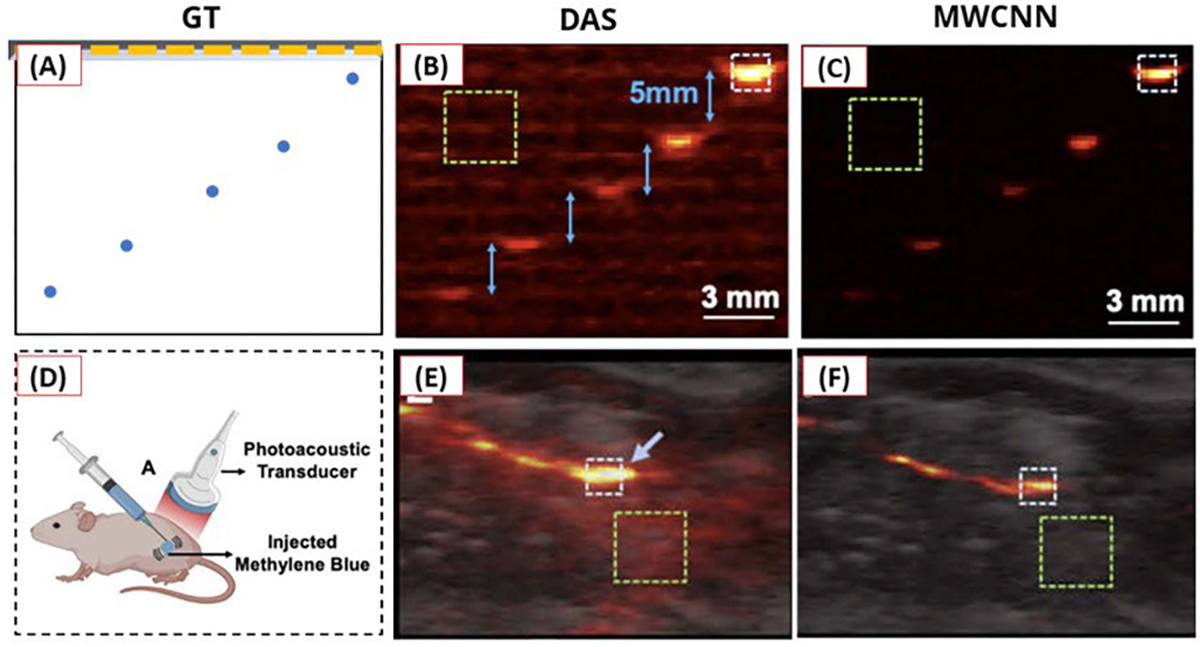
Reconstruction results using (a)–(c) in vitro phantom (pencil lead) data and (d)–(f) in vivo data acquired from methylene blue injected into a mouse. (a) and (d) Ground-truth or acquisition illustrations. (b) and (e) Standard image reconstruction results. (c) and (f) Results of the DL model fed by (b) and (e) images. All figures are reproduced with permission from [98].

To simulate complex vascular networks, they constructed an in vitro phantom using 3-D printing with a light-absorbing material. Additionally, TiO2-based optical scatters were introduced into the phantom to acquire low-fluence data typical of in vivo conditions. PA images from scattering and nonscattering media using a 15-MHz linear transducer served as training input and reference data, respectively. The authors employed a multilevel wavelet-CNN architecture, which was also based on the U-Net backbone. Common pooling operations in the U-Net architecture, typically employed to enlarge the receptive field, often result in irreversible information loss [105], [106]. Therefore, in this study, these pooling operations were replaced with discrete wavelet and inverse-wavelet transforms, gradually restoring image resolution to access multiscale features. As illustrated in Fig. 12, the DL model provided higher contrast-to-noise ratio (CNR) images compared to input images in in vivo experiments involving mice injected with contrast agents.

**Fig. 13.**
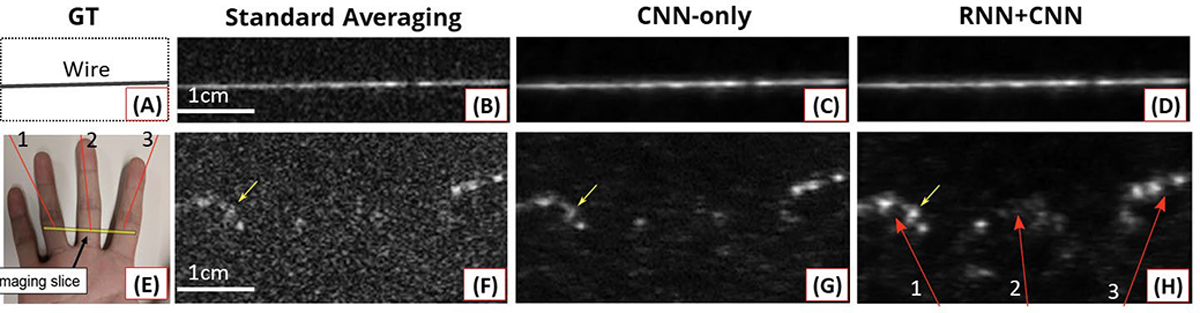
Reconstruction results using (a)–(d) in vitro phantom (wire) data and (e)–(h) in vivo data acquired from a human hand. Multiple frame images (DAS images) were averaged. (a) and (e) Ground-truth or acquisition illustrations. (b) and (f) Standard averaging results. (c) and (g) Results using only the CNN-based model. (d) and (h) Results using the proposed DL model combining CNN and RNN. All figures are reproduced with permission from [99].

A common approach to improve image quality in LED systems is to average multiple image frames, taking advantage of fast data acquisition. Anas et al. [99] proposed a DL model that effectively combines low-quality images to generate an enhanced image. The DL model integrated CNNs and RNNs, where the CNN extracted spatial features from each image frame and the RNN combined these features by considering temporal dependencies. To train and test the model, images were acquired from an in vitro phantom containing gold magnetic nanoparticles or wires. A reference image was generated by averaging multiple frames and filtering noise. The results demonstrated that the DL model produced clearer images compared to standard averaging techniques, as depicted in Fig. 13.

**Fig. 14.**
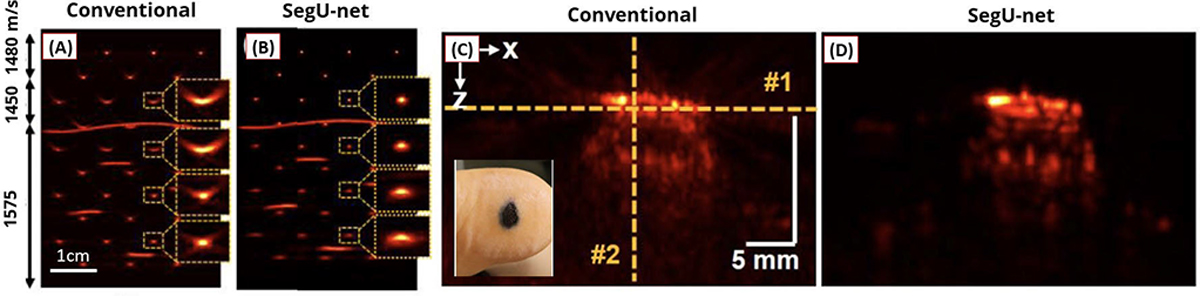
Reconstruction results using (a) and (b) in vitro phantom data and (c) and (d) in vivo data acquired from melanoma on a patient’s heel. The phantom is heterogeneous, where three layers had different SOSs. (a) and (c) Standard DAS images using 1540 m/s as SOS. (b) and (d) Results using the proposed DL method. All figures are reproduced with permission from [100].

The studies reviewed above assumed a static and known SOS in tissue. However, discrepancies between the assumed value and the actual value can lead to noticeable phase aberrations. While TR is a well-known technique for aberration correction, its main practical challenge lies in its limitation to coherent reflectors, such as a point target at the focal point [107]. In their research, Jeon et al. [100] proposed a UNET-based model named SegU-net to accurately determine the true static SOS and minimize aberration artifacts. The model was trained using multichannel images, each reconstructed using raw data and a different SOS within the range of 1460–1600 m/s. A PA image obtained using the true SOS served as the ground truth. These authors modified the UNET architecture by incorporating additional links between the encoder and decoder to facilitate detailed feature extraction. Both in-silico and in vivo experiments demonstrated that the network effectively enhanced the main lobe while suppressing sidelobes, reducing aberration artifacts even in heterogeneous media as illustrated in Fig. 14.

## IV. Conclusion and Discussion

### A. PAUS Imaging

PAUS imaging systems integrate a fiber-optic delivery system within a conventional clinical US transducer, enabling simultaneous PA and US imaging with flexible physical manipulation for human subject scanning. They can potentially image microvascular structures and blood perfusion in localized areas, leveraging the strong optical absorption of blood for contrast and high US frequencies for fine spatial resolution. Consequently, integrated PAUS imaging has the potential to detect and quantify vascular diseases such as atherosclerosis or stroke, as well as monitor angiogenesis. With a few cm light penetration depth (approximately 3–5 cm, depending on the optical scattering and absorption of background tissue for a specific application), PAUS imaging may also be well suited to image different forms of cancer, such as melanoma, ovarian, thyroid, muscle carcinoma, and breast cancers [21], [108].

Real-time PAUS imaging may also bring molecular sensitivity to conventional US. Spectroscopic PA imaging leverages the optical absorption spectrum of molecules to identify specific species in the body. Endogenous molecular imaging primarily exploits the molecular characteristics of hemoglobin and, under controlled conditions, can measure the local oxygenation state of blood [61]. Exogenous molecular imaging exploits the specific absorption spectrum of contrast agents for a range of applications in molecular diagnostics and therapy. Of particular interest are applications where molecular labeling is combined with real-time spectroscopic PAUS to guide interventions such as drug delivery, surgeries, and therapies [109].

Although the promise of PAUS imaging is substantial, the poor image quality of PA images reconstructed using the limited view and bandwidth of handheld US arrays has severely limited clinical adoption. As discussed in Section II, PA imaging to determine initial PA pressure (or heat function) serves as a crucial preliminary step before subsequent quantification of target absorbers. However, as evidenced by simulation results (see Fig. 4) for this acquisition geometry, scanning areas encompassing numerous absorbers produce PA images with significant artifacts and shape-distortions using conventional approaches. These images are not accurate and do not faithfully depict the distribution of initial PA pressure. Here, we have presented a review of diverse DL frameworks focused on overcoming these limitations of clinical PAUS imaging with a handheld probe.

### B. DL Reconstructions in PAUS Imaging

In particular, DL techniques can mitigate the physical limitations imposed by the PAUS platform, potentially translating this important tool into clinical applications. As demonstrated in Section II, PA image reconstruction (i.e., the acoustic inverse problem) is ill-posed for limited-view and limited-bandwidth data. To address this issue, target absorbers were inevitably constrained to point-like, small circular, or vascular objects. However, conventional methods like DAS or TR still produced low-contrast and low-resolution images with artifacts. The typical DL framework serves as a postprocessing tool, taking a conventionally reconstructed image (DAS or TR) as input and producing a higher quality output image. In particular, the UNET model is commonly chosen for this problem because both the input and output belong to the image domain and share the same size. In the model, encoder and decoder components are explicitly designed to extract and combine multiscale features, respectively.

To further enhance image quality, numerous studies utilizing the UNET framework extended the model’s access to additional information and modified the network structure to accommodate these changes. Additional information includes channel data, tensor data derived from channel data, combinations of channel data and a DAS image, or DAS images obtained using different sound speeds. In some cases, the model incorporates additional skip connections between the encoder and decoder or replaces existing skip connections with convolutional layers. These modifications access features at various levels of abstraction, enabling the network to leverage both low-level and high-level features for a more comprehensive representation.

In addition, alternative frameworks such as RNN or GAN have also been explored. For instance, one study combined RNN with CNN to address multiframe images and strategically average them. Another study adopted a GAN architecture, where the UNET-based generator aimed to generate images that closely resembled reference images by attempting to deceive the discriminator beyond standard loss metrics like mse. These novel frameworks offer additional avenues to improve PAUS image quality.

As the size of input data increases, there is a growing need for enhanced DL network efficiency. In line with this, as discussed in Section III, several research groups have incorporated a simple attention mechanism into the UNET architecture to capture spatial dependencies beyond the limitations of a convolutional filter [84]. Currently, the vision transformer (ViT) [110], which leverages the concept of self-attention, has performed at a high level for various medical imaging tasks by effectively capturing long-range dependencies. Unlike convolutional architectures, self-attention can capture relationships between different subsections in the full image domain regardless of their positions [111]. The ViT model is clearly a viable option for complex and voluminous PA data.

The majority of papers reviewed here focused on supervised approaches where in-silico data served as a reference for model training. However, the inherent discrepancy between in-silico training data and real test data is a major issue for clinical translation. Acquiring a large volume of ground-truth data (gold standards) is challenging, leading research groups to rely on synthetic data to train their models. Consequently, performance in a clinical setting heavily depends on the similarity between synthetic and real tissue models. To mitigate this issue, plausible ranges or distributions of optical parameter values for each tissue have been used, with the aim of facilitating successful transfer learning.

Despite these efforts, unexpected artifacts have been reported in many papers due to the underlying disparities between synthetic and real data. This is exemplified in Figs. 9 and 10, where the yellow arrows indicate the likelihood of these artifacts occurring. One presumable cause for these artifacts could be that the synthetic model is based on a 2-D geometry, while real data represent a 3-D environment. As discussed in Section II, the influence of absorbers extends beyond the plane of interest (in-plane) to those located out-of-plane in PA data acquisition. Given that each transducer element has elevational directivity during acquisition, misinterpreting PA signals from out-of-plane sources as originating from in-plane sources could be a plausible explanation for such artifacts. Thus, the development of more realistic 3-D tissue and transducer models holds the potential to bridge the gap between training and test data, leading to artifact reduction.

An alternative that can address this challenge is unsupervised learning using approaches such as CycleGAN [112]. CycleGAN uses GANs for image-to-image translation or raw data-to-image translation without requiring paired training data. The fundamental idea behind CycleGAN is cycle consistency loss, which forces the translated image to be accurately reconstructed back to its original form. For instance, in the case of translating a horse image to a zebra image to deceive a discriminator, the translated zebra image is not random but rather constrained to closely resemble the original image due to the loss. In the field of medical imaging, CycleGAN learned mappings between different domains, such as CT and MRI [113], enabling image transfer while preserving content. In the context of PA imaging, where obtaining paired data is challenging, exploiting CycleGAN can provide a more convenient approach for image reconstruction tasks.

PAUS systems offer a significant advantage by providing both a standard US image and a PA image [114]. This implies that DL networks can leverage US data to enhance PA imaging using acoustic features. For instance, US B-mode images can be particularly valuable in obtaining accurate SOS measurements. In a tissue domain with large vessels, US data can provide essential information about their position and shape, which may not be readily observed in PA images. In cases where involuntary movement occurs during scanning or data acquisition, US speckle can be used for motion tracking and compensation [26]. Furthermore, two different domains, US and PA, present a promising opportunity for unsupervised learning such as CycleGAN, offering considerable enhancements in real-time PAUS images. They have the potential to strengthen PA/US dual-mode imaging [115], [116], [117], offering complementary information that can enhance its translation into practical clinical applications.

### C. Extension of DL Frameworks

DL frameworks can be extended to further reinforce PAUS imaging. First, they can potentially mitigate clutter signals caused by sound reverberation between reflectors in tissue. For instance, Allman et al. [118] presented a CNN-based model designed to identify reflection artifacts and source signals, focusing on scenarios where the object was limited to point-like targets. In the domain of standard US imaging, numerous DL techniques have been introduced to suppress reverberation clutter [119], [120], [121].

Second, as 3-D US imaging evolves, free-hand PA imaging with DL can be extended to include 3-D imaging capabilities. In traditional US imaging, a 2-D matrix probe is employed to simultaneously scan multiple image planes and acquire volumetric data in real-time. However, this approach requires a higher cost to achieve high spatial resolution, and the computational demands are intense. One approach to tackle these challenges is the use of a sparse 2-D matrix probe, in which elements are intentionally skipped or spaced further apart to reduce the number of physical elements in the probe. In this context, DL plays a crucial role in enhancing image reconstruction from undersampled data, thus compensating for element reductions compared to dense arrays [122]. Additionally, panoramic imaging techniques can help create extended 3-D field-of-view images by stitching together multiple 2-D images obtained by sweeping a standard probe. Presently, DL methods were employed to estimate the probe’s position and movement without requiring any additional positional sensors [123], [124].

Finally, DL frameworks hold the potential to enhance spectroscopic PA imaging, mapping chromophore concentration using PA spectra acquired at different wavelengths. Accurately estimating chromophore concentrations poses a significant challenge due to spectral distortion [61], [125]. The PA spectrum at a specific spatial position is influenced not only by the linear combination of intrinsic absorption spectra of chromophores at that position but also by wavelength-varying optical fluence. DL methods [126], [127], [128], [129], [130] offered a solution to this challenge by mapping from PA images acquired at multiple wavelengths, eliminating the need for prior knowledge such as fluence maps and intrinsic absorption spectra of chromophores. Additionally, spectroscopic PA imaging holds promise for automatic segmentation and isolation of target objects. Currently, multispectral imaging combined with DL techniques improved task performance [129], [131], [132].

Although these methods have been validated in simulation settings, comprehensive validation in vivo remains largely unexplored. Presently, tissue phantom models used to generate training data are often considered overly simplistic to adequately simulate real-world scenarios. To tackle this limitation, some researchers have embarked on alternate approaches, such as creating phantoms that integrate information from other imaging modalities. For instance, Yang and Gao [128] designed a 3-D heterogeneous tissue structure by leveraging an MR breast database, subsequently assigning optical and acoustic parameters to replicate a more realistic environment. In existing literature, PA images reconstructed using DAS or TR methods are commonly used as input to DL models [126], [127], [128], [129], [130]. Expanding upon this framework, two approaches will be pursued: 1) using two separate DL networks, where one network focuses on generating clear PA images from PA signals, while the other network is dedicated to generating the map of chromophore concentration from PA images and 2) alternatively, a single DL network will be trained using an end-to-end learning approach, directly mapping PA signals to the predicted concentration map.

### D. Challenges

A current challenge to DL approaches for PA reconstruction is the lack of performance comparisons between DL methods in the current literature, mainly due to individual model optimization using separate datasets. To address this issue, the availability of a publicly accessible framework to evaluate novel DL methods using identical reference data, including both phantom and real data, would prove invaluable. This framework would facilitate the comparison of different methods, thereby expediting the advancement of PA reconstruction techniques. Open frameworks have already been established in some medical imaging fields to address similar challenges. For instance, the Challenge on Ultrasound Beamforming with DL (CUBDL) was offered during the 2020 IEEE International Ultrasonics Symposium [133].

Additionally, a significant challenge is in vivo validation of all DL techniques. This presents significant hurdles in translating these techniques to clinical practice and, to date, none have achieved clinical translation. While many DL studies have been validated in simulation by quantifying mse-based metrics between estimates and the ground-truth of initial PA pressure, their validation has often been limited to in vitro phantom experiments. The focus of these studies to date has been to assess the clarity of reconstructed absorber shapes and the reduction of artifacts arising from ill-posed conditions. Although some studies have extended validation to in vivo scenarios, these evaluations mainly focus on the plausibility of reconstructed absorber shapes, which relies heavily on prior anatomical knowledge. Given the scarcity of quantitative ground-truth maps in vivo, most papers have omitted quantitative evaluation of their DL results. For instance, in situations where the target is blood vessels, the initial PA pressure can exhibit positional variations within vessels, a factor often overlooked in evaluations. Therefore, a compelling future challenge involves addressing this limitation and developing methodologies to quantify the performance of DL techniques in vivo.

### E. Conclusion

In conclusion, reconstructing PAUS images with DL is very new, but there is no doubt that this framework holds significant potential to improve the modality. With the advent of next-generation computing systems, more complex and realistic tissue models can be created through simulations in a shorter time. The development of efficient networks trained on large volumes of data will greatly facilitate successful transfer learning from virtual environments to real-world applications. Additionally, unsupervised techniques, especially those incorporating US data, can potentially improve performance and make DL systems more robust for real clinical applications. Similar to recent advances in CT and MR imaging resulting from DL tools, as PAUS systems and imaging techniques become more standardized, a wealth of patient data will become available. Abundant human subject data will provide ample opportunities to thoroughly evaluate DL methods, leading to increased trust and confidence in their clinical utility.

## Figures and Tables

**TABLE I:** Data Acquisition Conditions and Reconstruction Models in Reviewed Papers

	Input Data	Backbone DL Model	Specialty in DL	Loss Function	DL Performance*	Training Data	In-vivo Test Data	Transducer (Center freq., bandwidth)
Waibel *et al.* [27]	Channel data or DAS image	UNET	Skip connection is replaced by conv. layer	MAE(Ll)	PSNR(DL)-PSNR(DAS) > 19.8 dB	In-silico Phantom	-	Linear (No ref.)
Lan *et al.* [96]	Channel data and DAS image	UNET	Both raw data and image are inputted.	MSE(L2)	PSNR(DL)-PSNR(DAS) > 7.8 dB	In-silico Phantom	Human palm vessels	Linear (7MHz, 80%)
Kim *et al.* [37]	Transformed data	UNET	Transformed data are inputted	MSE(L2)	PSNR(DL)-PSNR(DAS) > 6.7 dB	In-silico Phantom	Human finger vessels	Linear (15MHz, 53%)
Vu *et al.* [97]	TR image	CGAN	Conditional GAN is used	MSE(L2) + Wasser-stein	PSNR(GAN)-PSNR(UNET) > 0.8 dB	In-silico Phantom	Mouse trunk vessels	Linear (5MHz, 60%)
Hariri *et al.* [98]	DAS image	UNET	Pooling step is replaced by Wavelet transform	MSE(L2)	PSNR(DL)-PSNR(DAS) > 1.9 dB	In-vitro Phantom	Contrast agent injected into mouse	Linear (15MHz, No ref.)
Anas *et al.* [99]	DAS images (multi-frame)	CNN+RNN	Muti-frame images are inputted	MSE(L2)	PSNR(DL)-PSNR(DAS averaging) > 5.9dB	In-vitro Phantom	Human finger vessels	Linear (No ref.)
Jeon *et al.* [100]	DAS images (multi-SOS)	UNET	More links between encoder and decoder	MSE(L2)	SNR_dB_(DL)-SNR_dB_(DAS) > 20dB	In-silico Phantom	Melanoma on a human subject’s heel	Linear (8.5MHz, No ref.)

* Improvement in PSNR/SNR with respect to the standard method in simulation/in-vitro tests

**TABLE II:** Equations and Parameters for Simulation

Circular array	Aperture length Element pitch Element numbers Radius Center frequency 3 dB bandwidth	125.6 mm 0.2 mm 628 20 mm 15.63 MHz 8 MHz
Linear array	Aperture length Element pitch Element numbers Center frequency 3 dB bandwidth	25.6 mm 0.1 mm 256 15.63 MHz 8 MHz
Data generation	Applied equation Absorbed energy in each object (H(r)) System function (ψ()) Noise $\left(n(t,r_{j}^{\prime })\right)$	Eqs. 5 & 6 1 (constant) Identity function 0
Reconstruction	Applied equation	Eq. 8

## Highlights

- Integrated PA and US (PAUS) imaging holds promise for disease detection and interventions, yet clinical adoption is hindered by poor image quality from handheld US arrays.
- Numerous studies highlight the potential of deep learning techniques to overcome PAUS platform limitations, but challenges are the lack of performance comparisons and in vivo validations.
- New networks may further enhance performance by successful transfer learning from more complex virtual tissue model to real applications or unsupervised learning incorporating US data.

## Appendix Reconstruction Simulation

We employed a custom simulation method outlined in (5) and (6) for data generation and (8) for image reconstruction. To simplify the simulation, in (6), the system function was set as an identity function, and the acquisition noise was set to zero. As depicted in Fig. 4(a), absorbed energy in each object [H(r) in (5)] remained constant. For both the circular array and linear array, the center frequency was 15.63 MHz, and the 3-dB bandwidth was 8 MHz. Table II summarizes all parameter values.

## References

[1] McDonald FA and Wetsel GC Jr., “Generalized theory of the photoacoustic effect,” J. Appl. Phys, vol. 49, no. 4, pp. 2313–2322, Apr. 1978.

[2] Xu M and Wang LV, “Photoacoustic imaging in biomedicine,” Rev. Sci. Instrum, vol. 77, no. 4, Apr. 2006, Art. no. 041101.

[3] Beard P, “Biomedical photoacoustic imaging,” Interface Focus, vol. 1, pp. 602–631, Aug. 2011. 22866233 10.1098/rsfs.2011.0028 PMC3262268

[4] Manwar R, Kratkiewicz K, and Avanaki K, “Overview of ultrasound detection technologies for photoacoustic imaging,” Micromachines, vol. 11, no. 7, p. 692, Jul. 2020. 32708869 10.3390/mi11070692 PMC7407969

[5] Wang LV, “Prospects of photoacoustic tomography,” Med. Phys, vol. 35, no. 12, pp. 5758–5767, Dec. 2008. 19175133 10.1118/1.3013698 PMC2647010

[6] Su JL , “Advances in clinical and biomedical applications of photoacoustic imaging,” Expert Opinion Med. Diag, vol. 4, no. 6, pp. 497–510, Nov. 2010. 10.1517/17530059.2010.529127 PMC3041963 21344060

[7] Li L, Li S, Fan Z, Huang G, Tang J, and Nie L, “Current strategies of photoacoustic imaging assisted cancer theragnostics toward clinical studies,” ACS Photon, vol. 9, no. 8, pp. 2555–2578, Aug. 2022.

[8] Rao AP, Bokde N, and Sinha S, “Photoacoustic imaging for management of breast cancer: A literature review and future perspectives,” Appl. Sci, vol. 10, no. 3, p. 767, Jan. 2020.

[9] Weber J, Beard PC, and Bohndiek SE, “Contrast agents for molecular photoacoustic imaging,” Nature Methods, vol. 13, no. 8, pp. 639–650, Aug. 2016. 27467727 10.1038/nmeth.3929

[10] Han S, Lee D, Kim S, Kim H-H, Jeong S, and Kim J, “Contrast agents for photoacoustic imaging: A review focusing on the wavelength range,” Biosensors, vol. 12, no. 8, p. 594, Aug. 2022. 36004990 10.3390/bios12080594 PMC9406114

[11] Attia ABE , “A review of clinical photoacoustic imaging: Current and future trends,” Photoacoustics, vol. 16, Dec. 2019, Art. no. 100144. 31871888 10.1016/j.pacs.2019.100144 PMC6911900

[12] Jeon S, Kim J, Lee D, Baik JW, and Kim C, “Review on practical photoacoustic microscopy,” Photoacoustics, vol. 15, Sep. 2019, Art. no. 100141. 31463194 10.1016/j.pacs.2019.100141 PMC6710377

[13] Erfanzadeh M and Zhu Q, “Photoacoustic imaging with low-cost sources; a review,” Photoacoustics, vol. 14, pp. 1–11, Jun. 2019. 30923674 10.1016/j.pacs.2019.01.004 PMC6423351

[14] Upputuri PK and Pramanik M, “Fast photoacoustic imaging systems using pulsed laser diodes: A review,” Biomed. Eng. Lett, vol. 8, no. 2, pp. 167–181, May 2018. 30603201 10.1007/s13534-018-0060-9 PMC6208528

[15] Nyayapathi N and Xia J, “Photoacoustic imaging of breast cancer: A mini review of system design and image features,” Proc. SPIE, vol. 24, no. 12, 2019, Art. no. 121911. 10.1117/1.JBO.24.12.121911 PMC7005545 31677256

[16] Choi W, Park E-Y, Jeon S, and Kim C, “Clinical photoacoustic imaging platforms,” Biomed. Eng. Lett, vol. 8, no. 2, pp. 139–155, May 2018. 30603199 10.1007/s13534-018-0062-7 PMC6208525

[17] Wang LV, “Multiscale photoacoustic microscopy and computed tomography,” Nature Photon, vol. 3, no. 9, pp. 503–509, Sep. 2009. 10.1038/nphoton.2009.157 PMC2802217 20161535

[18] Na S and Wang LV, “Photoacoustic computed tomography for functional human brain imaging,” Biomed. Opt. Exp, vol. 12, no. 7, pp. 4056–4083, 2021. 10.1364/BOE.423707 PMC8367226 34457399

[19] Gu Y, Sun Y, Wang X, Li H, Qiu J, and Lu W, “Application of photoacoustic computed tomography in biomedical imaging: A literature review,” Bioeng. Transl. Med, vol. 8, no. 2, Mar. 2023, Art. no. e10419. 36925681 10.1002/btm2.10419 PMC10013779

[20] Yang J, Choi S, and Kim C, “Practical review on photoacoustic computed tomography using curved ultrasound array transducer,” Biomed. Eng. Lett, vol. 12, pp. 19–35, Feb. 2022. 35186358 10.1007/s13534-021-00214-8 PMC8825902

[21] Steinberg I, Huland DM, Vermesh O, Frostig HE, Tummers WS, and Gambhir SS, “Photoacoustic clinical imaging,” Photoacoustics, vol. 14, pp. 77–98, Jun. 2019. 31293884 10.1016/j.pacs.2019.05.001 PMC6595011

[22] Kratkiewicz K, Pattyn A, Alijabbari N, and Mehrmohammadi M, “Ultrasound and photoacoustic imaging of breast cancer: Clinical systems, challenges, and future outlook,” J. Clin. Med, vol. 11, no. 5, p. 1165, Feb. 2022. 35268261 10.3390/jcm11051165 PMC8911419

[23] Zhou Y, Yao J, and Wang LV, “Tutorial on photoacoustic tomography,” J. Biomed. Opt, vol. 21, no. 6, Apr. 2016, Art. no. 061007. 27086868 10.1117/1.JBO.21.6.061007 PMC4834026

[24] Xia J, Yao J, and Wang LV, “Photoacoustic tomography: Principles and advances,” Prog. Electromagn. Res, vol. 147, pp. 1–22, 2014. 10.2528/pier14032303 PMC4311576 25642127

[25] Xu M and Wang LV, “Universal back-projection algorithm for photoacoustic computed tomography,” Phys. Rev. E, Stat. Phys. Plasmas Fluids Relat. Interdiscip. Top, vol. 71, no. 1, Jan. 2005, Art. no. 016706. 10.1103/PhysRevE.71.016706 15697763

[26] Jeng G-S , “Real-time interleaved spectroscopic photoacoustic and ultrasound (PAUS) scanning with simultaneous fluence compensation and motion correction,” Nature Commun, vol. 12, no. 1, pp. 1–12, Jan. 2021. 33514737 10.1038/s41467-021-20947-5 PMC7846772

[27] Waibel D, Gröhl J, Isensee F, Kirchner T, Maier-Hein K, and Maier-Hein L, “Reconstruction of initial pressure from limited view photoacoustic images using deep learning,” Proc. SPIE, vol. 10494, pp. 196–203, Feb. 2018.

[28] Kim J, Lee D, Jung U, and Kim C, “Photoacoustic imaging platforms for multimodal imaging,” Ultrasonography, vol. 34, no. 2, pp. 88–97, Apr. 2015. 25754364 10.14366/usg.14062 PMC4372714

[29] Kim J , “Programmable real-time clinical photoacoustic and ultrasound imaging system,” Sci. Rep, vol. 6, no. 1, pp. 1–11, Oct. 2016. 27731357 10.1038/srep35137 PMC5059665

[30] Nam SY and Emelianov SY, “Array-based real-time ultrasound and photoacoustic ocular imaging,” J. Opt. Soc. Korea, vol. 18, no. 2, pp. 151–155, Apr. 2014.

[31] Montilla LG, Olafsson R, Bauer DR, and Witte RS, “Real-time photoacoustic and ultrasound imaging: A simple solution for clinical ultrasound systems with linear arrays,” Phys. Med. Biol, vol. 58, no. 1, pp. N1–N12, Jan. 2013. 23221479 10.1088/0031-9155/58/1/N1

[32] Zhu Y , “Towards clinical translation of LED-based photoacoustic imaging: A review,” Sensors, vol. 20, no. 9, p. 2484, Apr. 2020. 32349414 10.3390/s20092484 PMC7249023

[33] Hariri A, Lemaster J, Wang J, Jeevarathinam AS, Chao DL, and Jokerst JV, “The characterization of an economic and portable led-based photoacoustic imaging system to facilitate molecular imaging,” Photoacoustics, vol. 9, pp. 10–20, Mar. 2018. 29234601 10.1016/j.pacs.2017.11.001 PMC5723278

[34] Wei C-W , “Real-time integrated photoacoustic and ultrasound (PAUS) imaging system to guide interventional procedures: Ex vivo study,” IEEE Trans. Ultrason., Ferroelectr., Freq. Control, vol. 62, no. 2, pp. 319–328, Feb. 2015. 25643081 10.1109/TUFFC.2014.006728 PMC4610852

[35] Bell MAL, “Photoacoustic imaging for surgical guidance: Principles, applications, and outlook,” J. Appl. Phys, vol. 128, no. 6, Aug. 2020, Art. no. 060904. 32817994 10.1063/5.0018190 PMC7428347

[36] Schellenberg MW and Hunt HK, “Hand-held optoacoustic imaging: A review,” Photoacoustics, vol. 11, pp. 14–27, Sep. 2018. 30073147 10.1016/j.pacs.2018.07.001 PMC6068331

[37] Kim M, Jeng G-S, Pelivanov I, and O’Donnell M, “Deep-learning image reconstruction for real-time photoacoustic system,” IEEE Trans. Med. Imag, vol. 39, no. 11, pp. 3379–3390, Nov. 2020. 10.1109/TMI.2020.2993835 PMC8594135 32396076

[38] Ai M , “Investigation of photoacoustic tomography reconstruction with a limited view from linear array,” J. Biomed. Opt, vol. 26, no. 9, Sep. 2021, Art. no. 096009. 34585543 10.1117/1.JBO.26.9.096009 PMC8477256

[39] Kremkau FW and Taylor KJ, “Artifacts in ultrasound imaging,” J. Ultrasound Med, vol. 5, no. 4, pp. 227–237, Apr-1986. 3514956 10.7863/jum.1986.5.4.227

[40] Rau R, Schweizer D, Vishnevskiy V, and Goksel O, “Ultrasound aberration correction based on local speed-of-sound map estimation,” in Proc. IEEE Int. Ultrason. Symp. (IUS), Oct. 2019, pp. 2003–2006.

[41] Park S, Karpiouk AB, Aglyamov SR, and Emelianov SY, “Adaptive beamforming for photoacoustic imaging using linear array transducer,” in Proc. IEEE Ultrason. Symp., Nov. 2008, pp. 1088–1091. 10.1364/ol.33.001291 PMC2713818 18552935

[42] Deán-Ben XL and Razansky D, “Portable spherical array probe for volumetric real-time optoacoustic imaging at centimeter-scale depths,” Opt. Exp, vol. 21, no. 23, pp. 28062–28071, 2013. 10.1364/OE.21.028062 24514320

[43] Karthikesh MS and Yang X, “Photoacoustic image-guided interventions,” Exp. Biol. Med, vol. 245, no. 4, pp. 330–341, Feb. 2020. 10.1177/1535370219889323 PMC7370598 31747782

[44] John S , “Niche preclinical and clinical applications of photoacoustic imaging with endogenous contrast,” Photoacoustics, vol. 32, Aug. 2023, Art. no. 100533. 37636547 10.1016/j.pacs.2023.100533 PMC10448345

[45] LeCun Y, Bengio Y, and Hinton G, “Deep learning,” Nature, vol. 521, no. 7553, pp. 436–444, 2015. 26017442 10.1038/nature14539

[46] Guo Y, Liu Y, Oerlemans A, Lao S, Wu S, and Lew MS, “Deep learning for visual understanding: A review,” Neurocomputing, vol. 187, pp. 27–48, Apr. 2016.

[47] Shinde PP and Shah S, “A review of machine learning and deep learning applications,” in Proc. 4th Int. Conf. Comput. Commun. Control Autom. (ICCUBEA), Aug. 2018, pp. 1–6.

[48] van der Laak J, Litjens G, and Ciompi F, “Deep learning in histopathology: The path to the clinic,” Nature Med, vol. 27, no. 5, pp. 775–784, May 2021. 33990804 10.1038/s41591-021-01343-4

[49] Lee D, Lee J, Ko J, Yoon J, Ryu K, and Nam Y, “Deep learning in MR image processing,” Investigative Magn. Reson. Imag, vol. 23, no. 2, pp. 81–99, 2019.

[50] Wang S, Xiao T, Liu Q, and Zheng H, “Deep learning for fast MR imaging: A review for learning reconstruction from incomplete k-space data,” Biomed. Signal Process. Control, vol. 68, Jul. 2021, Art. no. 102579.

[51] Domingues I, Pereira G, Martins P, Duarte H, Santos J, and Abreu PH, “Using deep learning techniques in medical imaging: A systematic review of applications on CT and PET,” Artif. Intell. Rev, vol. 53, no. 6, pp. 4093–4160, Aug. 2020.

[52] McLeavy CM , “The future of CT: Deep learning reconstruction,” Clin. Radiol, vol. 76, no. 6, pp. 407–415, Jun. 2021. 33637310 10.1016/j.crad.2021.01.010

[53] Kawaguchi K, Kaelbling LP, and Bengio Y, “Generalization in deep learning,” 2017, arXiv:1710.05468.

[54] Deng H, Qiao H, Dai Q, and Ma C, “Deep learning in photoacoustic imaging: A review,” J. Biomed. Opt, vol. 26, no. 4, Apr. 2021, Art. no. 040901. 33837678 10.1117/1.JBO.26.4.040901 PMC8033250

[55] Gröhl J, Schellenberg M, Dreher K, and Maier-Hein L, “Deep learning for biomedical photoacoustic imaging: A review,” Photoacoustics, vol. 22, Jun. 2021, Art. no. 100241. 33717977 10.1016/j.pacs.2021.100241 PMC7932894

[56] Rajendran P, Sharma A, and Pramanik M, “Photoacoustic imaging aided with deep learning: A review,” Biomed. Eng. Lett, vol. 12, pp. 155–173, May 2022. 35529338 10.1007/s13534-021-00210-y PMC9046497

[57] Yang C, Lan H, Gao F, and Gao F, “Review of deep learning for photoacoustic imaging,” Photoacoustics, vol. 21, Mar. 2021, Art. no. 100215. 33425679 10.1016/j.pacs.2020.100215 PMC7779783

[58] Yang C, Lan H, Gao F, and Gao F, “Deep learning for photoacoustic imaging: A survey,” 2020, arXiv:2008.04221.

[59] Wang LV and Wu H-I, Biomedical Optics: Principles and Imaging. Hoboken, NJ, USA: Wiley, 2012.

[60] Gusev VE and Karabutov AA, “Laser optoacoustics,” NASA STI/RECON, Washington, DC, USA, Tech. Rep 93, 1991.

[61] Cox B, Laufer JG, Arridge SR, and Beard PC, “Quantitative spectroscopic photoacoustic imaging: A review,” J. Biomed. Opt, vol. 17, no. 6, 2012, Art. no. 061202. 22734732 10.1117/1.JBO.17.6.061202

[62] Jacques SL, “Optical properties of biological tissues: A review,” Phys. Med. Biol, vol. 58, no. 11, pp. R37–R61, Jun. 2013. 23666068 10.1088/0031-9155/58/11/R37

[63] Zhou X, Akhlaghi N, Wear KA, Garra BS, Pfefer TJ, and Vogt WC, “Evaluation of fluence correction algorithms in multispectral photoacoustic imaging,” Photoacoustics, vol. 19, Sep. 2020, Art. no. 100181. 32405456 10.1016/j.pacs.2020.100181 PMC7210453

[64] Grasso V, Holthof J, and Jose J, “An automatic unmixing approach to detect tissue chromophores from multispectral photoacoustic imaging,” Sensors, vol. 20, no. 11, p. 3235, Jun. 2020. 32517204 10.3390/s20113235 PMC7308815

[65] Xu Y, Xu M, and Wang LV, “Exact frequency-domain reconstruction for thermoacoustic tomography. II. Cylindrical geometry,” IEEE Trans. Med. Imag, vol. 21, no. 7, pp. 829–833, Jul. 2002. 10.1109/TMI.2002.801171 12374320

[66] Cox BT, Arridge SR, and Beard PC, “Photoacoustic tomography with a limited-aperture planar sensor and a reverberant cavity,” Inverse Problems, vol. 23, no. 6, pp. S95–S112, Dec. 2007.

[67] Lin X, Yu J, Feng N, and Sun M, “Synthetic aperture-based lineararray photoacoustic tomography considering the aperture orientation effect,” J. Innov. Opt. Health Sci, vol. 11, no. 4, Jul. 2018, Art. no. 1850015.

[68] Tang Y , “High-fidelity deep functional photoacoustic tomography enhanced by virtual point sources (conference presentation),” Proc. SPIE, vol. PC12379, Mar. 2023, Art. no. PC1237912. 10.1016/j.pacs.2023.100450 PMC9852650 36685991

[69] Xu M and Wang LV, Universal Back-Projection Algorithm for Photoacoustic Tomography. Boca Raton, FL, USA: CRC Press, 2017, pp. 37–46.

[70] Szabo TL, Diagnostic Ultrasound Imaging: Inside Out. New York, NY, USA: Academic, 2004.

[71] Liang D, Zhang HF, and Ying L, “Compressed-sensing photoacoustic imaging based on random optical illumination,” Int. J. Funct. Informat. Pers. Med, vol. 2, no. 4, pp. 394–406, 2009.

[72] Zhang C, Zhang Y, and Wang Y, “A photoacoustic image reconstruction method using total variation and nonconvex optimization,” Biomed. Eng. OnLine, vol. 13, no. 1, pp. 1–29, Dec. 2014. 25129644 10.1186/1475-925X-13-117 PMC4148921

[73] Ding L, Deán-Ben XL, and Razansky D, “Real-time model-based inversion in cross-sectional optoacoustic tomography,” IEEE Trans. Med. Imag, vol. 35, no. 8, pp. 1883–1891, Aug. 2016. 10.1109/TMI.2016.2536779 26955023

[74] Han Y, Ding L, Ben XLD, Razansky D, Prakash J, and Ntziachristos V, “Three-dimensional optoacoustic reconstruction using fast sparse representation,” Opt. Lett, vol. 42, no. 5, pp. 979–982, 2017. 28248347 10.1364/OL.42.000979

[75] Måsøy S-E , “Aberration correction in 2D echocardiography,” Quant. Imag. Med. Surg, vol. 13, no. 7, pp. 4603–4617, Jul. 2023. 10.21037/qims-22-895 PMC10347361 37456280

[76] Schwab H-M, Beckmann MF, and Schmitz G, “Iterative photoacoustic reconstruction in heterogeneous media using the Kaczmarz method,” in Proc. IEEE Int. Ultrason. Symp., Sep. 2014, pp. 33–36.

[77] Moradi H, Honarvar M, Tang S, and Salcudean SE, “Iterative photoacoustic image reconstruction for three-dimensional imaging by conventional linear-array detection with sparsity regularization,” Proc. SPIE, vol. 10064, pp. 510–514, Mar. 2017.

[78] Goodfellow I, Bengio Y, and Courville A, Deep Learning. Cambridge, MA, USA: MIT Press, 2016.

[79] Cunningham P, Cord M, and Delany SJ, Supervised Learning. Berlin, Germany: Springer, 2008, pp. 21–49.

[80] Voulodimos A, Doulamis N, Doulamis A, and Protopapadakis E, “Deep learning for computer vision: A brief review,” Comput. Intell. Neurosci, vol. 2018, pp. 1–13, Feb. 2018. 10.1155/2018/7068349 PMC5816885 29487619

[81] Alzubaidi L , “Review of deep learning: Concepts, CNN architectures, challenges, applications, future directions,” J. Big Data, vol. 8, no. 1, pp. 1–74, Mar. 2021. 33816053 10.1186/s40537-021-00444-8 PMC8010506

[82] Ronneberger O, Fischer P, and Brox T, “U-Net: Convolutional networks for biomedical image segmentation,” in Proc. Int. Conf. Med. Image Comput. Comput.-Assist. Intervent. Munich, Germany: Springer, 2015, pp. 234–241.

[83] Chan Y, Wavelet Basics. Norwell, MA, USA: Springer, 1994.

[84] Oktay O , “Attention U-Net: Learning where to look for the pancreas,” 2018, arXiv:1804.03999.

[85] Wang F , “Residual attention network for image classification,” in Proc. IEEE Conf. Comput. Vis. Pattern Recognit. (CVPR), Jul. 2017, pp. 3156–3164.

[86] Hu J, Shen L, and Sun G, “Squeeze-and-excitation networks,” in Proc. IEEE/CVF Conf. Comput. Vis. Pattern Recognit., Jun. 2018, pp. 7132–7141.

[87] Fan Y, Lu X, Li D, and Liu Y, “Video-based emotion recognition using CNN-RNN and C3D hybrid networks,” in Proc. 18th ACM Int. Conf. Multimodal Interact., Oct. 2016, pp. 445–450.

[88] Rehman A and Belhaouari SB, “Deep learning for video classification: A review,” TechRxiv, 2021.

[89] Goodfellow I , “Generative adversarial networks,” Commun. ACM, vol. 63, no. 11, pp. 139–144, 2020.

[90] Gui J, Sun Z, Wen Y, Tao D, and Ye J, “A review on generative adversarial networks: Algorithms, theory, and applications,” IEEE Trans. Knowl. Data Eng, vol. 35, no. 4, pp. 3313–3332, Apr. 2023.

[91] Bowles C , “GAN augmentation: Augmenting training data using generative adversarial networks,” 2018, arXiv:1810.10863.

[92] Frid-Adar M, Diamant I, Klang E, Amitai M, Goldberger J, and Greenspan H, “GAN-based synthetic medical image augmentation for increased CNN performance in liver lesion classification,” Neurocomputing, vol. 321, pp. 321–331, Dec. 2018.

[93] Liu H-B and Lee I, “MPL-GAN: Toward realistic meteorological predictive learning using conditional GAN,” IEEE Access, vol. 8, pp. 93179–93186, 2020.

[94] Isola P, Zhu J-Y, Zhou T, and Efros AA, “Image-to-image translation with conditional adversarial networks,” in Proc. IEEE Conf. Comput. Vis. Pattern Recognit. (CVPR), Jul. 2017, pp. 1125–1134.

[95] Mathieu M, Couprie C, and LeCun Y, “Deep multi-scale video prediction beyond mean square error,” 2015, arXiv:1511.05440.

[96] Lan H, Jiang D, Yang C, Gao F, and Gao F, “Y-Net: Hybrid deep learning image reconstruction for photoacoustic tomography in vivo,” Photoacoustics, vol. 20, Dec. 2020, Art. no. 100197. 32612929 10.1016/j.pacs.2020.100197 PMC7322183

[97] Vu T, Li M, Humayun H, Zhou Y, and Yao J, “A generative adversarial network for artifact removal in photoacoustic computed tomography with a linear-array transducer,” Exp. Biol. Med, vol. 245, no. 7, pp. 597–605, Apr. 2020. 10.1177/1535370220914285 PMC7153213 32208974

[98] Hariri A, Alipour K, Mantri Y, Schulze JP, and Jokerst JV, “Deep learning improves contrast in low-fluence photoacoustic imaging,” Biomed. Opt. Exp, vol. 11, no. 6, pp. 3360–3373, 2020. 10.1364/BOE.395683 PMC7316023 32637260

[99] Anas EMA, Zhang HK, Kang J, and Boctor E, “Enabling fast and high quality LED photoacoustic imaging: A recurrent neural networks based approach,” Biomed. Opt. Exp, vol. 9, no. 8, pp. 3852–3866, 2018. 10.1364/BOE.9.003852 PMC6191624 30338160

[100] Jeon S, Choi W, Park B, and Kim C, “A deep learning-based model that reduces speed of sound aberrations for improved in vivo photoacoustic imaging,” IEEE Trans. Image Process, vol. 30, pp. 8773–8784, 2021. 34665732 10.1109/TIP.2021.3120053

[101] Staal J, Abramoff MD, Niemeijer M, Viergever MA, and van Ginneken B, “Ridge-based vessel segmentation in color images of the retina,” IEEE Trans. Med. Imag, vol. 23, no. 4, pp. 501–509, Apr. 2004. 10.1109/TMI.2004.825627 15084075

[102] Gulrajani I, Ahmed F, Arjovsky M, Dumoulin V, and Courville AC, “Improved training of Wasserstein GANs,” in Proc. Adv. Neural Inf. Process. Syst, vol. 30, 2017, pp. 1–11.

[103] Li J, Madry A, Peebles J, and Schmidt L, “On the limitations of first-order approximation in GAN dynamics,” 2017, arXiv:1706.09884.

[104] Uhlirova H , “Neurovascular network explorer 2.0: A database of 2-photon single-vessel diameter measurements from mouse Si cortex in response to optogenetic stimulation,” Frontiers Neuroinform, vol. 11, p. 4, Feb. 2017. 10.3389/fninf.2017.00004 PMC5285378 28203155

[105] Xu G, Liao W, Zhang X, Li C, He X, and Wu X, “Haar wavelet downsampling: A simple but effective downsampling module for semantic segmentation,” Pattern Recognit., vol. 143, Nov. 2023, Art. no. 109819.

[106] Liu W, Yan Q, and Zhao Y, “Densely self-guided wavelet network for image denoising,” in Proc. IEEE/CVF Conf. Comput. Vis. Pattern Recognit. Workshops (CVPRW), Jun. 2020, pp. 432–433.

[107] Ali R , “Aberration correction in diagnostic ultrasound: A review of the prior field and current directions,” Zeitschrift Medizinische Physik, vol. 33, no. 3, pp. 267–291, Aug. 2023. 10.1016/j.zemedi.2023.01.003 PMC10517407 36849295

[108] Valluru KS and Willmann JK, “Clinical photoacoustic imaging of cancer,” Ultrasonography, vol. 35, no. 4, pp. 267–280, Oct. 2016. 27669961 10.14366/usg.16035 PMC5040138

[109] Xia J, Kim C, and Lovell J, “Opportunities for photoacoustic-guided drug delivery,” Current Drug Targets, vol. 16, no. 6, pp. 571–581, Jul. 2015. 26148989 10.2174/1389450116666150707100328 PMC5435469

[110] Han K , “A survey on vision transformer,” IEEE Trans. Pattern Anal. Mach. Intell, vol. 45, no. 1, pp. 87–110, Jan. 2023. 35180075 10.1109/TPAMI.2022.3152247

[111] Khan S, Naseer M, Hayat M, Zamir SW, Khan FS, and Shah M, “Transformers in vision: A survey,” ACM Comput. Surv, vol. 54, no. 10, pp. 1–41, Jan. 2022.

[112] Zhu J-Y, Park T, Isola P, and Efros AA, “Unpaired image-to-image translation using cycle-consistent adversarial networks,” in Proc. IEEE Int. Conf. Comput. Vis. (ICCV), Oct. 2017, pp. 2223–2232.

[113] Yang H , “Unpaired brain MR-to-CT synthesis using a structure-constrained CycleGAN,” in Deep Learning in Medical Image Analysis and Multimodal Learning for Clinical Decision Support. Granada, Spain: Springer, Sep. 2018, pp. 174–182.

[114] Agrawal S, Suresh T, Garikipati A, Dangi A, and Kothapalli S-R, “Modeling combined ultrasound and photoacoustic imaging: Simulations aiding device development and artificial intelligence,” Photoacoustics, vol. 24, Dec. 2021, Art. no. 100304. 34584840 10.1016/j.pacs.2021.100304 PMC8452892

[115] Wen Y , “Clinical photoacoustic/ultrasound dual-modal imaging: Current status and future trends,” Frontiers Physiol, vol. 13, p. 2227, Oct. 2022. 10.3389/fphys.2022.1036621 PMC9651137 36388111

[116] Oraevsky AA, Clingman B, Zalev J, Stavros AT, Yang WT, and Parikh JR, “Clinical optoacoustic imaging combined with ultrasound for coregistered functional and anatomical mapping of breast tumors,” Photoacoustics, vol. 12, pp. 30–45, Dec. 2018. 30306043 10.1016/j.pacs.2018.08.003 PMC6172480

[117] Lee H, Choi W, Kim C, Park B, and Kim J, “Review on ultrasound-guided photoacoustic imaging for complementary analyses of biological systems in vivo,” Exp. Biol. Med, vol. 248, no. 9, pp. 762–774, May 2023. 10.1177/15353702231181341 PMC10468641 37452700

[118] Allman D, Reiter A, and Bell MAL, “Photoacoustic source detection and reflection artifact removal enabled by deep learning,” IEEE Trans. Med. Imag, vol. 37, no. 6, pp. 1464–1477, Jun. 2018. 10.1109/TMI.2018.2829662 PMC6075868 29870374

[119] Brickson LL, Hyun D, and Dahl JJ, “Reverberation noise suppression in the aperture domain using 3D fully convolutional neural networks,” in Proc. IEEE Int. Ultrason. Symp. (IUS), Oct. 2018, pp. 1–4. 10.1109/TMI.2021.3049307 PMC8500501 33400649

[120] Brickson LL, Hyun D, Jakovljevic M, and Dahl JJ, “Reverberation noise suppression in ultrasound channel signals using a 3D fully convolutional neural network,” IEEE Trans. Med. Imag, vol. 40, no. 4, pp. 1184–1195, Apr. 2021. 10.1109/TMI.2021.3049307 PMC8500501 33400649

[121] Luchies A and Byram B, “Suppressing off-axis scattering using deep neural networks,” Proc. SPIE, vol. 10580, pp. 84–91, Mar. 2018.

[122] Lok U-W , “Deep variational network for high quality 3D ultrasound imaging using sparse array,” in Proc. IEEE Int. Ultrason. Symp. (IUS), Sep. 2020, pp. 1–4.

[123] Guo H, Xu S, Wood B, and Yan P, “Sensorless freehand 3D ultrasound reconstruction via deep contextual learning,” in Medical Image Computing and Computer Assisted Intervention—MICCAI 2020. Lima, Peru: Springer, Oct. 2020, pp. 463–472.

[124] Prevost R , “3D freehand ultrasound without external tracking using deep learning,” Med. Image Anal, vol. 48, pp. 187–202, Aug. 2018. 29936399 10.1016/j.media.2018.06.003

[125] Kim M, Jeng G-S, O’Donnell M, and Pelivanov I, “Correction of wavelength-dependent laser fluence in swept-beam spectroscopic photoacoustic imaging with a hand-held probe,” Photoacoustics, vol. 19, Sep. 2020, Art. no. 100192. 32670789 10.1016/j.pacs.2020.100192 PMC7339128

[126] Cai C, Deng K, Ma C, and Luo J, “End-to-end deep neural network for optical inversion in quantitative photoacoustic imaging,” Opt. Lett, vol. 43, no. 12, pp. 2752–2755, Jun. 2018. 29905680 10.1364/OL.43.002752

[127] Yang C, Lan H, Zhong H, and Gao F, “Quantitative photoacoustic blood oxygenation imaging using deep residual and recurrent neural network,” in Proc. IEEE 16th Int. Symp. Biomed. Imag. (ISBI), Apr. 2019, pp. 741–744.

[128] Yang C and Gao F, “EDA-Net: Dense aggregation of deep and shallow information achieves quantitative photoacoustic blood oxygenation imaging deep in human breast,” in Proc. Int. Conf. Med. Image Comput. Comput.-Assist. Intervent. Shenzhen, China: Springer, 2019, pp. 246–254.

[129] Bench C, Hauptmann A, and Cox B, “Toward accurate quantitative photoacoustic imaging: Learning vascular blood oxygen saturation in three dimensions,” J. Biomed. Opt, vol. 25, no. 8, Aug. 2020, Art. no. 085003. 32840068 10.1117/1.JBO.25.8.085003 PMC7443711

[130] Gröhl J, Kirchner T, Adler T, and Maier-Hein L, “Estimation of blood oxygenation with learned spectral decoloring for quantitative photoacoustic imaging (LSD-qPAI),” 2019, arXiv:1902.05839.

[131] Luke GP, Hoffer-Hawlik K, Van Namen AC, and Shang R, “O-Net: A convolutional neural network for quantitative photoacoustic image segmentation and oximetry,” 2019, arXiv:1911.01935.

[132] Schellenberg M , “Semantic segmentation of multispectral photoacoustic images using deep learning,” Photoacoustics, vol. 26, Jun. 2022, Art. no. 100341. 35371919 10.1016/j.pacs.2022.100341 PMC8968659

[133] Bell MAL, Huang J, Hyun D, Eldar YC, van Sloun R, and Mischi M, “Challenge on ultrasound beamforming with deep learning (CUBDL),” in Proc. IEEE Int. Ultrason. Symp. (IUS), Sep. 2020, pp. 1–5.

